# Optimizing predictive models for evaluating the *F*-temperature index in predicting the *π*-electron energy of polycyclic hydrocarbons, applicable to carbon nanocones

**DOI:** 10.1038/s41598-024-72896-w

**Published:** 2024-10-26

**Authors:** Sakander Hayat, Muhammad Yasir Hayat Malik, Seham J. F. Alanazi, Saima Fazal, Muhammad Imran, Muhammad Azeem

**Affiliations:** 1 Mathematical Sciences, Faculty of Science, Universiti Brunei Darussalam, Jln Tungku Link, Gadong, BE1410 Brunei Darussalam Brunei; 2 Department of Mathematics, Division of Science and Technology, University of Education, Lahore, Pakistan; 3 Department of Chemistry, College of Science (CS), King Saud University, Riyadh, 11451 Saudi Arabia; 4 Department of Mathematical Sciences, College of Science, United Arab Emirate University Al Ain 15551, Abu Dhabi, UAE; 5 Department of Solids and Structures, School of Engineering, The University of Manchester, Oxford Road, Manchester, M13 9PL UK; 6 Department of Mathematics, Riphah International University, Lahore, Pakistan

**Keywords:** Mathematical chemistry, Discrete optimization model, Temperature-based graphical index, Structure-property model, Total $\pi$-electron energy, Carbon nanocone, Cheminformatics, Physical chemistry, Applied mathematics

## Abstract

In the fields of mathematics, chemistry, and the physical sciences, graph theory plays a substantial role. Using modern mathematical techniques, quantitative structure-property relationship (QSPR) modeling predicts the physical, synthetic, and natural properties of substances based only on their chemical composition. For a chemical graph, the temperature of a vertex is a local property introduced by Fajtlowicz (1988). A temperature-based graphical descriptor is structured based on temperatures of vertices. Involving a non-zero real parameter $\beta$, the general *F*-temperature index $T_{\beta }$ is a temperature index having strong efficacy. In this paper, we employ discrete optimization and regression analysis to find optimal value(s) of $\beta$ for which the prediction potential of $T_{\beta }$ and the total $\pi$-electron energy $E_{\pi }$ of polycyclic hydrocarbons is the strongest. This, in turn, answers an open problem proposed by Hayat & Liu (2024). Applications of the optimal values for $T_{\beta }$ are presented a two-parametric family of carbon nanocones in predicting their $E_{\pi }$ with significantly higher accuracy.

## Introduction

Chemical graph compatibility is mostly determined by topology. Topological indices have found extensive use across various fields, including chemistry, drug design, biological activity prediction, and environmental risk assessment. These indices are predictive of many physicochemical properties, including toxicity, surface tension, boiling point, viscosity, dipole moment, aqueous solubility, and refractive index. Investigating fundamental ideas in chemistry requires extensive use of mathematical computations. Numerous studies in this area have been conducted during the past few decades. Chemical graph theory and mathematical chemistry use molecular graphs, also called chemical graphs, as graph theory representations of the structural formula of chemical compounds. A labeled graph with edges denoting chemical bonds and vertices designating the atoms in the particle is called a chemical graph. On its edges are labels indicating the types of bonds, and on its vertices are the types of connected atoms.

If $\vartheta$ and *H* are both isomorphic, then $T(\vartheta )= T(H)$ is a topological index. Specifically, *T* is a function $T:\varphi \rightarrow {\mathbb {R}}$, where ${\mathbb {R}}$ (resp. $\varphi$) the set of real numbers (resp. set of simple connected graphs). Note that, for a graph $H=(V_H,E_H)$, its order $n=|V_H|$ and size $m=|E_H|$ also map the graph *H* to positive real numbers, and thus delivering some trivial examples of topological indices. To create regression models that correlate the physicochemical, biological, or thermodynamic aspects of chemical substances, structure-property modeling uses molecular descriptors^1^. An effective way to correlate the physicochemical features of benzenoid hydrocarbons (BHs) is through the use of degree-based graphical indices, a class of graph-theoretic molecular descriptors. In research examining the relationship between quantitative structure and property (QSPR) and quantitative structure and activity (QSAR), this index has been the most used molecular descriptor over time. A brief summary of its mathematical features may be found in two recent monographs. These have undergone considerable examination. Furthermore, in the scientific literature, an array of modifications and alternative formulations of this index have been proposed (see^2,3^). For a current overview of the structure-property modeling of the physicochemical characteristics of nanostructures and biomolecular networks, see^4–10^. Cruz et al.^11^ studied benzenoid systems with a small number of inlets in 2013.

To assess the quality of a given class of molecular graphical descriptors, a comparison analysis using appropriate test molecules and their specific chemical properties is usually conducted. The validity of degree-dependent graphical descriptors for correlating the physicochemical properties of isomeric octanes, which are typical of alkanes, was examined by Gutman & Tošović^12^. This research on degree-based molecular indices was expanded to include benzenoid hydrocarbons (BHs) from octane-isomers by Malik et al.^13^. More work was done on the quantum-theoretical (resp. thermodynamic) characteristics of BHs by Hayat et al.^14^ (resp. Hayat et al.^15^). Isomeric octanes were chosen by Gutman & Tošović^12^ as test molecules in their work, although lower 20-30 BHs were used as text molecules in other investigations^12,13,15^. As an alternative, Hayat et al.^14^ chose the total $\pi$-electronic energy ($E_{\pi }$) to reflect quantum-theoretical features, and Hayat et al.^15^ chose the entropy and heat capacity to support thermodynamic attributes.

The quality testing noted earlier indicated that the first general temperature $T^1_\alpha$ and second general temperature $T^2_\alpha$ indices had a great potential to effectively correlate the physicochemical, thermodynamic, and quantum-theoretical features of benzenoid hydrocarbons. Hayat et al.^14^ demonstrated that $SCI_{-\frac{1}{2}}$ & $R_{0.2661}$ are the most effective descriptors for predicting $E_{\pi }$ of BHs, whereas Hayat et al.^15^ investigated the best two indices for correlating thermodynamic properties of BHs, which are $SCI_{-3}$ & $R_{-1}$. The drawback of these studies is that they only take into account $R_{\alpha }$ and $SCI_{\alpha }$ for finite values of $\alpha$, that is, $\alpha \in \left\{ \pm \frac{1}{2},\pm 1,\pm 2\right\}$. The generic $\alpha \in {\mathbb {R}}\setminus \{0\}$ should be taken into consideration while examining $R_{\alpha }$ and $SCI_{\alpha }$ as they both have a high potential to connect different features of BHs.

The molecular topology plays a crucial role in the electrical configurations of two-dimensional structures like carbon nanocones and three-connected carbon systems including 1*D* and 2*D* nanotubes. The manufacture of highly oriented pyrolytic graphite, graphene, carbon nanotubes, various forms of carbon films, and carbon electrodes for screen printing are the main topics of this comprehensive review. It also discusses new characteristics, unique carbon materials, new synthetic techniques, and electroanalytical uses for carbon materials^16^. In 1994, Carbon nanocones (CNCs) were initially seen by Ge and Sattler^17^, which are formations with centered five-member rings. The 60 wedge is carved out using a graphene sheet, and the edges come together to form a cone with a single pentagonal flaw at the apex. The analysis of carbon cones of nanometer size was conducted using scanning tunneling microscopy (STM) after carbon atoms were produced through vapor condensation on a graphite substrate.

Owing to its potential usage in a wide range of applications, such as chemical probes, gas sensors, biosensors, energy storage, and nano electronic devices, carbon nanocones have drawn a lot of attention^18^. It has been suggested that carbon allotropes like carbon nanotubes and nanocones could be used as molecular gas storage devices. Because of their special qualities and probable functions in a wide range of inventive domains, incorporating storing gas and production, carbon nanocones have attracted more attention from scientists in recent times^19^. The PI and edge szeged indices of one-heptagonal carbon nanocones were ascertained by Ashrafi et al.^20^ in 2009. One-heptagonal carbon nanocones’ Wiener index can be calculated numerically using the approach developed by Alipour et al.^21^. An individual pentagonal carbon nanocone’s eccentric connectivity index was examined by Saheli et al.^22^. The issue of calculating one pentagonal carbon nanocone’s eccentric connection index was discussed in this study.

The topological characteristics of $CNC_{k}[n]$ nanocones were studied by Hayat et al.^23^ in 2014. The $GA_4$, $GA_5$ and *ABC* indices for conical graphite were calculated in this article. Furthermore, they demonstrated the two significant partition types of $CNC_{k}[n]$ nanocones in two parameters *k* and *n*. Carbon nanohorns (or nanocones) are conical carbon nanostructures composed of a $sp_2$ carbon sheet, as defined in 2016. Nanohorns can be made in large quantities and don’t require a metal catalyst during their creation. A novel method for disecting these “dahlia-like” clusters into individual nanocones has been developed to get around this restriction^25^. The *GA* indices of nanocones $CNC_{k}[n]$ and the edges version of *ABC* were the areas of operation for Gao et al.^24^. The purpose of this work was to further investigate the *ABCe* and *GAe* indices of these conical graphites. The edge-based version of the geometric arthmetic index of 8-cycle polyomino links and stochastic nanocones was utilized by Zahid Raza^26^ in the same year. This research was investigated the index of molecular topology based on degrees, or the more advanced version of GA, with regard to carbon nanocones and 2-polyomino chains with 8 circles, an intriguing class of carbon nanomaterials.

**Fig. 1:**
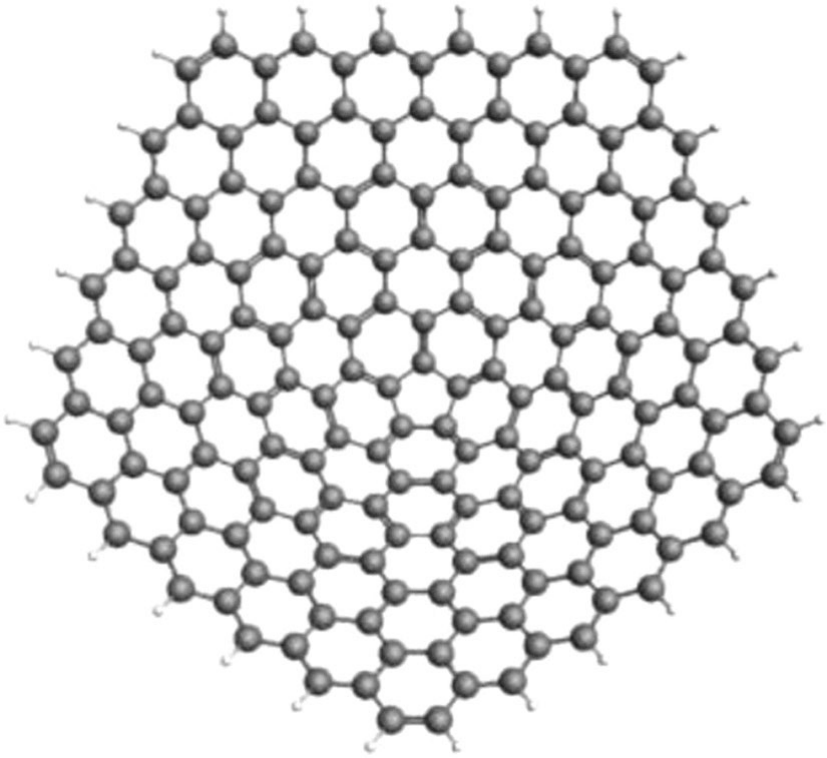
A pentagonal carbon nanocone. Generated by employing HyperChem^32^.

The study conducted in 2018 by Hayat et al.^27^ examined the applications of valency-based topological descriptors of chemical networks. The researchers computed precise equations for the well-performing indices of several enormous groups of carbon nanotubes, cones, and a recently introduced kinship among biological networks known as diamond structures in tetrahedral geometry. Hussain^28^ discussed the computational features of the carbon nanocone line graph. They derived exact formulations for M-polynomials of line graphs with nanocones. The study conducted by García-Hernández et al.^29^ concerns the theoretical analysis of the *CO*, $CO_2$, and $NO_2$ acquisition on carbon nanocones that are pristine or doped with BN. Bultheel^30^ discussed the topology of carbon nanocones made of 1-pentagons, as well as geometrical relevance and magical sizes. In order to obtain significant findings about desired sizes and chemical reactivity, topological modeling techniques were utilized in this paper to examine one pentagon carbon nanocones. See Figure 1, for a 3D visualization of a pentagonal nanocone. For a detailed study of the electronic structure of carbon pentagonal nancones, we refer to^31^.

In 2019, Kulli^33^ published a paper regarding multiplicative F-indices and their connectivity F-indices of chemical networks. The multiplicative (first, second), first and second hyper, sum connectivity, product connectivity, atom bond connectivity and geometric-arithmetic F-index of three types of polyhex nanotubes: armchair, zigzag, and carbon nanocone networks were all calculated in this study. The *Q* operator of a carbon nanocone’s SK, F-topological, and hyper Zagreb index were determined by Lokesha et al.^34^. Utilizing topological indices makes it simple to comprehend the physical characteristics of these structures. In this paper, they used the *Q* operator to construct the *SK*, *F*, *S*, and Carbon nanocones’ hyper-Zagreb index. The research investigation conducted by Arockiaraj et al.^35^ focused on utilizing topological indices constructed around edge distance of strength-weighted graphs for coronoid systems, carbon nanocones, and $SiO_2$ nanostructures.

Muhammad Shoaib et al.^36^ in 2020 accurately determined the harmonic indices and harmonic polynomials of carbon nanocones $CNC_{k}[n]$. Haidar et al.^37^ worked on topological characteristics of the triangle sheet of boron *BTS* for *m* and *n*, the Melem string *MC*(*n*), and the Borophene string $B_{36}(m)$ in 2022. This study explored and provided analytical closed results for the general Randic index $R_a(G)$. For sheet and chain applications, they also computed the generic $M_1$, *ABC*, *GA*, $ABC_4$, and $GA_5$ indices for the first time and provided closed formulas for these degree-based indices. The formulations for nanostructures using topological indices were recently analyzed by Ying et al.^38^.

In this study, we find the value(s) of $\alpha$ for which $R_{\alpha }$ and $SCI_{\alpha }$ have substantial prediction potential for BH physicochemical features. Regression analysis and correlation analysis have also been performed to determine the optimal $\alpha$ with the highest correlation. Also we calculate the graphical indices for carbon nanocones $CNC_{k}[n]$, which are based on temperature. We’ll now talk about a few fundamental definitions that will help us in our work.

## Preliminaries

From the corpus of recent literature, a few essential terms and definitions are examined here.

### Definition 2.1

In 1988, Fajtlowicz^39^ transmitted the following notation for the temperature $T_b$ of a vertex *b* of a *n*-vertex graph:

1 $$\begin{aligned} T_b = \frac{d_b}{n-d_b} \end{aligned}$$

where, in the graph $\vartheta$, *n* and $d_b$ indicate the total number of vertices and the degree of vertex *b*, respectively. The temperature-based graphical index $\vartheta _t$ for a chemical graph $\vartheta =(V_\vartheta ,E_\vartheta )$ has the generic form $\vartheta _t=\sum \limits _{rs\in E_G}\psi (T_r,T_s)$, where $\psi$ is a symmetric map and $T_i$ is the temperature of $i\in V_\vartheta$.

A class of molecular descriptors used in chemical graph theory that are based on the temperature of vertices is known as temperature-based topological indices. Kulli^40^ in 2019 presented the temperature-based indices. The following are well-known temperature indices:

- First Hyper Temperature Index
  The $HT_1$ index is defined as,
  2 $$\begin{aligned} HT_1(\vartheta )=\sum _{edges}(T_r+T_s)^2. \end{aligned}$$
  In the graph $\vartheta$, $T_r$ and $T_s$ denotes the temperature index of vertex *r* and *s* respectively. The generic temperature index $T^\sigma _1$ generalizes the first hyper temperature index by considering a parameter $\sigma \in R$.
  3 $$\begin{aligned} T^\sigma _1(\vartheta )=\sum _{edges} (T_r+ T_s)^\sigma , \end{aligned}$$
  we have $HT_1=T^\sigma _1$ with $\sigma = 2$.
- Second Hyper Temperature Index
  The $HT_2$ index is defined as,
  4 $$\begin{aligned} HT_2(\vartheta )=\sum _{edges}(T_r T_s)^2. \end{aligned}$$
  The generic temperature index $T^\sigma _2$ generalizes the second hyper temperature index by considering a parameter $\sigma \in R$.
  5 $$\begin{aligned} T^\sigma _2(\vartheta )=\sum _{edges} (T_r T_s)^\sigma , \end{aligned}$$
  we have $HT_2=T^\sigma _2$ with $\sigma = 2$.
- Sum-Connectivity Temperature Index
  The *ST* index is stated as,
  6 $$\begin{aligned} ST(\vartheta )=\sum _{edges} \frac{1}{\sqrt{T_r+T_s}} = \sum _{edges} (T_r+T_s)^{-\frac{1}{2}}. \end{aligned}$$
  The generic temperature index $T^\sigma _1$ for a parameter $\sigma \in R$ is a generalization of the sum-connectivity temperature index.
  7 $$\begin{aligned} T^\sigma _1(\vartheta )=\sum _{edges} (T_r+T_s)^\sigma , \end{aligned}$$
  we have $ST = T^\sigma _1$ with $\sigma = -\frac{1}{2}$.
- Temperature Index of Product-Connectivity
  The *PT* index for a graph $\vartheta$ is stipulated as,
  8 $$\begin{aligned} PT(\vartheta )=\sum _{edges} \frac{1}{\sqrt{T_r T_s}} = \sum _{edges} (T_r T_s)^{-\frac{1}{2}}. \end{aligned}$$
  For a parameter $\sigma \in R$, the product-connectivity temperature index can be extended to the generic temperature index $T^\sigma _2$.
  9 $$\begin{aligned} T^\sigma _2(\vartheta )=\sum _{edges} (T_r T_s)^\sigma , \end{aligned}$$
  we have $PT = T^\sigma _2$ with $\sigma = \frac{-1}{2}$.
- The Temperature Index for Reciprocal Product-Connectivity
  For a graph $\vartheta$, the *RPT* index is specified as,
  10 $$\begin{aligned} RPT(\vartheta )=\sum _{edges} \sqrt{T_r T_s} = \sum _{edges} (T_r T_s)^{\frac{1}{2}}. \end{aligned}$$
  The *RPT* index is generalized by the general temperature index $T^\sigma _2$ for a parameter $\sigma \in R$.
  11 $$\begin{aligned} T^\sigma _2(\vartheta )=\sum _{edges} (T_r T_s)^\sigma , \end{aligned}$$
  we have $RPT = T^\sigma _2$ with $\sigma = \frac{1}{2}$.
- Arithmetic-Geometric Temperature Index
  In the case of a graph $\vartheta$, the *AGT* index is defined by,
  12 $$\begin{aligned} AGT(\vartheta )=\sum _{edges} \left( \frac{T_r+T_s}{2\sqrt{T_r T_s}}\right) . \end{aligned}$$
- Forgotten temperature Index
  For a given graph $\vartheta$, the *FT* index may be written like this:
  13 $$\begin{aligned} FT(\vartheta )=\sum _{edges}(T_r^2+ T_s^2). \end{aligned}$$
  The generic temperature index $T_\sigma$ with a parameter $\sigma \in R$ generalizes the F-temperature index.
  14 $$\begin{aligned} T_\sigma (\vartheta )=\sum _{edges}(T_r^\sigma + T_s^\sigma ), \end{aligned}$$
  we have $FT = T_\sigma$ with $\sigma =2$.
- Temperature Sombor Index
  The *TSO* index is based on the Somber degree descriptor and was initially created by Gutman^41^. The construction of the TSO index is as follows:
  15 $$\begin{aligned} TSO(\vartheta )= \sum _{edges}\sqrt{T_{r}^{2}+ T_{s}^{2}}. \end{aligned}$$
- Modified Temperature Sombor Index
  Kulli^42^ developed the $^{m}TSO$ index and determined the precise analytical repression of $^{m}TSO$ for certain types of nanotubes. It is described as
  16 $$\begin{aligned} ^{m}TSO(\vartheta )= \sum _{edges}\frac{1}{\sqrt{T_r^2+ T_s^2}}. \end{aligned}$$
- Harmonic Temperature Index
  Harmonic temperature index is based on harmonic valency descriptor was proposed and studied by Narayankar et al.^43^ in 2018. Its definition is given below with regard to a chemical graph $\vartheta$.
  17 $$\begin{aligned} HT(\vartheta )= \sum _{edges}\frac{2}{T_r+ T_s}. \end{aligned}$$
- Geometric-Arithmetic Temperature Index
  The *GAT* index is defined as,
  18 $$\begin{aligned} GAT(\vartheta )=\sum _{edges} \left( \frac{2\sqrt{T_r T_s}}{T_r+T_s}\right) . \end{aligned}$$
- Reduced Reciprocal Product-Connectivity Temperature Index
  Hayat & Liu^44^ presented two new graphical temperature indices. The following formula yields the *RRPT* index for a graph $\vartheta$:
  19 $$\begin{aligned} RRPT(\vartheta )= \sum _{edges}\sqrt{(T_r-1)(T_s-1)}. \end{aligned}$$
- Atom-bond Connectivity(ABC) Temperature Index
  The *ABCT* index was initially investigated by Kahsay et al.^45^ for certain carbon nanostructures. It is described as
  20 $$\begin{aligned} ABCT(\vartheta )= \sum _{edges} \sqrt{\left| \frac{T_r+ T_s-2}{T_r T_s}\right| }. \end{aligned}$$

### Definition 2.2

The correlation coefficient between two finite-mean random variables *X* and *Y* is defined as follows in the field of statistics: $$\rho _{XY} = {{\text {cov}}}(X,Y)/\sigma _X\sigma _Y \in [-1,1]$$. The covariance function is denoted by $${\text {cov}}$$, and the standard deviations of the random variables *X* and *Y* are represented by $$\sigma _X$$ and $$\sigma _Y$$, respectively. A *predictorY* and a response variable *X* have a linear connection, and the correlation coefficient quantifies both the direction and intensity of this relationship. In the case of $$x_n$$ and $$y_n$$$$(n = 1, 2, \ldots , k)$$, a sequence of *k* measurements of these variables, the value $$\rho _{XY}$$ is calculated by

21 $$\begin{aligned} r_{xy} = \frac{\sum ^k_{n=1}(x_n-{\bar{x}})(y_n-{\bar{y}})}{\sqrt{\sum ^k_{n=1}(x_n-{\bar{x}})^2}\sqrt{\sum ^k_{n=1}(y_n-{\bar{y}})^2}}, \end{aligned}$$

where $\bar{x} = \frac{1}{k}\sum ^k_{n=1}x_n$ and $\bar{y} = \frac{1}{k}\sum ^k_{n=1}y_n$. Strong linear relationships between *X* and *Y* are indicated by $|\rho _{XY}|$ values that are closer to 1.

By assuming a *regression line*$Y = aX + b + \epsilon$, where $\epsilon$ represents random errors and $\{a,b \in {\mathbb {R}}\}$ are coefficients to be estimated, the correlation coefficient is closely related to the idea of *linear regression* of *Y* versus *X*. Typically, the *ordinary least squares* approach is used; closed-form solutions of the estimators ${\hat{a}}$ and ${\hat{b}}$ for *a* and *b*, respectively, are well-known and easily accessible. Specifically, ${\hat{a}} = r_{xy} s_y / s_x$ for this *simple linear regression* model, where $s_x$ and $s_y$ represent the unbiased estimators of $\sigma _x$ and $\sigma _Y$, respectively, and ${\hat{b}} = {\bar{y}} - {\hat{a}} {\bar{x}}$. It appears that the regression line’s slope and the correlation are connected.

### Definition 2.3

Two important goodness-of-fit metrics in regression analysis are the correlation coefficient and the *standard error of fit*. One definition of the standard error of fit is

22 $$\begin{aligned} s(Y,X) = \sqrt{\frac{1}{k-2}\sum ^k_{n=1}(y_n - y'_n)^2}, \end{aligned}$$

where the projected value coming from the regression line is $y'_n = {\hat{a}} x_n + {\hat{b}}$. This measures the degree to which the observed values differ from the values that the model anticipated. They can be computed using different statistical or mathematical applications.

### Definition 2.4

**Fig. 2:**
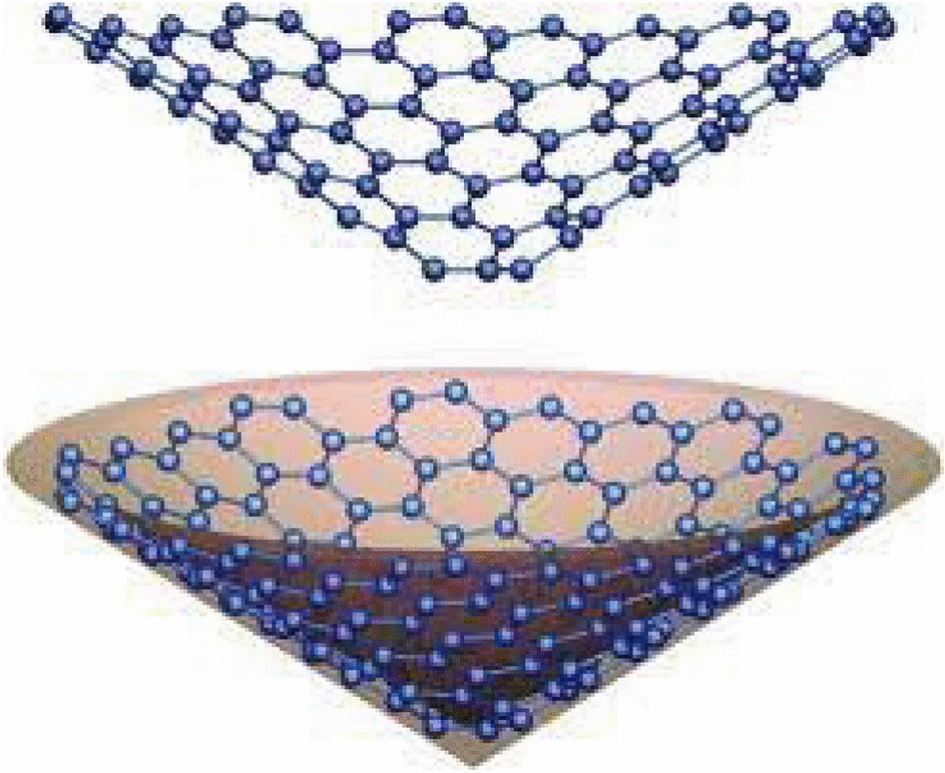
Conical shape of a pentagonal carbon nanocone. Adopted from^46^.

The graphical structure of carbon nanocones $CNC_{k}[n], k\ge 3, n\in N$ is composed of a *k*-length cycle at its core and *n*-levels of hexagons at the conical boundary around the center of the structure. The $CNC_{k}[n]$ has $k(1+2n +\sum _{i=1}^{n}(3i-1))$ edges and $k\sum _{i=1}^{n+1}(2i-1)$ nodes in its molecular graph, Figure 9 illustrates this. Figure 2 presents a 3D conical shape of a pentagonal carbon nanocone.

### Definition 2.5

Edge partitioning divides a graph into numerous balanced divisions within a given size, hence reducing the number of vertices that need to be sliced. The disjoint partitioning of $\vartheta$’s edge set into *m* subsets $E_{k}$ is referred to as partitioning. Partitioning of $E_{k}\cap E_{l}= \emptyset$ and $E_{k}\subseteq E,\cup _{k\in [m]} E_{k} = E$ for each $k \ne l$. The carbon nanocone’s edge partitioning is displayed in table 5.

## Materials and methods

All benzenoid hydrocarbons may be naturally represented by benzenoid systems, which are finite linked planar graphs without cut vertices in which every internal face is surrounded by a regular hexagon with sides of unit length.

**Fig. 3:**
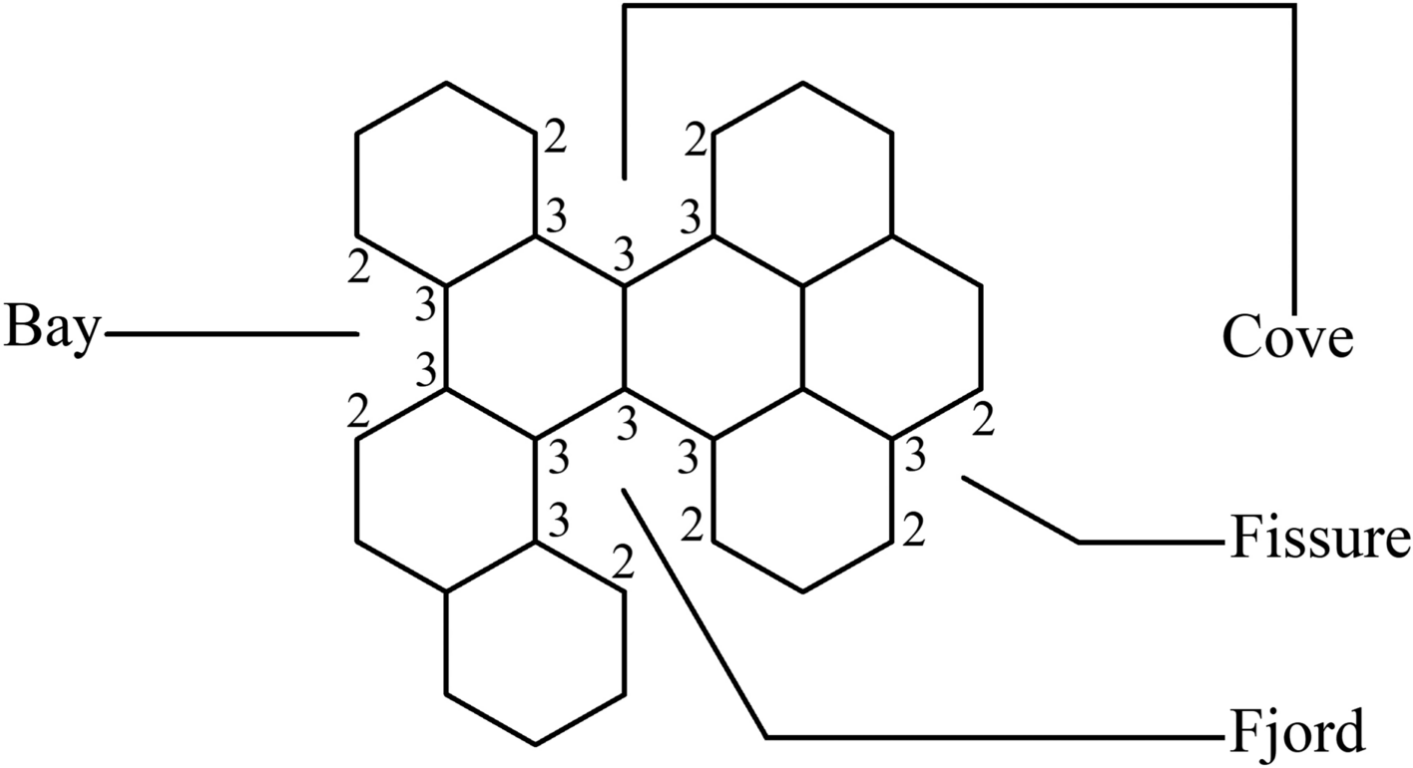
Fissure, cove, bay and fjord in a benzenoid system. The image is generated by using Mayura draw.

The definitions shown below are appropriate; they are taken from Assume that *B* is a benzenoid system with *p* hexagons and *v* vertices. The associated vertex degree sequence for each path $p_1 - p_2 - \cdots - p_{\ell +1}$ of length $\ell ~(\ell \in {\mathbb {N}}, \ell \ge 1$) within *B* is $(d_{p_1}, d_{p_2}, \ldots , d_{p_{\ell +1}})$. The pathways with degree sequences (2, 3, 3, 3, 3, 2), (2, 3, 3, 3, 2), (2, 3, 3, 2) and (2, 3, 2) are denoted by a *fjord*, *cove*, *bay*, and *fissure*, respectively. These pathways are followed all the way around *B*, as Figure 3 shows. Figure 3 was generated by the authors by using Mayura draw software. There are several varieties of *inlets*, including fjords, coves, bays, and fissures. So, the entire number of fjords, coves, bays, and fissures all up equals the number of inlets, or *k*.

Let us consider a benzenoid system *B* with *v* vertices, *k* inlets, and *p* hexagons. Let $n_{ij}$ represent the number of edges in *B* that satisfy the given requirements, where $d_a = i$ and $d_b = j$ are the degrees of an edge’s endpoints, *a* and *b*, respectively. By Lemma 1 in^47^, we have

23 $$\begin{aligned} n_{22} = v - 2p - k + 2, \quad n_{23} = 2k, \quad n_{33} = 3p - k - 3. \end{aligned}$$

By (14) and (23), the benzenoid system *B* has the the *F*-temperature indices are defines as follows:

24 $$\begin{aligned} \begin{aligned} T_{\beta }(G)&= \sum _{xy \in E} (T^{\beta }_x + T^{\beta }_y) \\&= n_{22} \left[ \left( \frac{2}{\nu - 2} \right) ^{\beta } + \left( \frac{2}{\nu - 2} \right) ^{\beta } \right] + n_{23} \left[ \left( \frac{2}{\nu - 2} \right) ^{\beta } + \left( \frac{3}{\nu - 3} \right) ^{\beta } \right] + n_{33} \left[ \left( \frac{3}{\nu - 3} \right) ^{\beta } + \left( \frac{3}{\nu - 3} \right) ^{\beta } \right] \\&= 2 \cdot (v - 2p - k + 2) \left[ \left( \frac{2}{\nu - 2} \right) ^{\beta } \right] + 2k \left[ \left( \frac{2}{\nu - 2} \right) ^{\beta } + \left( \frac{3}{\nu - 3} \right) ^{\beta } \right] + 2 \cdot (3p - k - 3) \left[ \left( \frac{3}{\nu - 3} \right) ^{\beta } \right] \\&= \frac{2^{\beta } (2v - 4p - 2k + 4)}{(\nu - 2)^{\beta }} + 2k \left[ \left( \frac{2}{\nu - 2} \right) ^{\beta } + \left( \frac{3}{\nu - 3} \right) ^{\beta } \right] + \frac{3^{\beta } (6p - 2k - 6)}{(\nu - 3)^{\beta }}. \end{aligned} \end{aligned}$$

In equations (24), *p*, *k*, and *v* represent the respective counts of hexagons, inlets, and vertices in the benzenoid system denoted as *B*, with $\alpha \in {\mathbb {R}}\setminus \{0\}$. We employ (24) to compute the $T_{\beta }$ for the 30 lower BHs given in Table 1.

The standard Pi Electronic Energy ($E_{\pi }$) and molecular structure of several polycyclic aromatic hydrocarbons (PAHs) are given in Table 1. Note that images in Table 1 were generated by using Mayura draw software. Table 2 also provides information on the *F*-temperature index $T_{\beta }$ of thirty lower benzenoid hydrocarbons.

**Table 1:** For thirty lower benzenoid hydrocarbons, the structural arrangement of the molecules and experimental information on the standard Pi Electronic Energy ($E_\Pi$) are given.

Molecule	Structure	$E_{\pi }$	Molecule	Structure	$E_{\pi }$
Benzene		8.0000	Benzo[g]chrysene		30.9990
Naphthalene		13.6832	Pentahelicene		30.9362
Anthracene		19.3137	Benzo[c]chrysene		30.9386
Phenanthrene		19.4483	Picene		30.9432
Tetracene		24.9308	Benzo[b]chrysene		30.8390
Benzo[c]phenanthrene		25.1875	Dibenzo[a,c]anthracene		30.9418
Benzo[a]anthracene		25.1012	Dibenzo[b,g]phenanthrene		30.8336
Chrysene		25.1922	Perylene		28.2453
Triphenylene		25.2745	Benzo[e]pyrene		28.3361
Pyrene		22.5055	Benzo[a]pyrene		28.2220
Pentacene		30.5440	Hexahelicene		36.6814
Benzo[a]tetracene		30.7255	Benzo[ghi]perylene		31.4251
Dibenzo[a,h]anthracene		30.8805	Hexacene		36.1557
Dibenzo[a,j]anthracene		30.8795	Coronene		34.5718
Pentaphene		30.7627	Ovalene		46.4974

**Table 2:** The *F*-temperature index $T_{\beta }$ of 30 lower benzenoid hydrocarbons.

Molecule	$T_{\beta }$
Benzene	$12 \cdot \left( \frac{1}{2} \right) ^\beta$
Napthalene	$12 \cdot \left( \frac{1}{4} \right) ^\beta + 4 \cdot \left[ \left( \frac{1}{4} \right) ^\beta + \left( \frac{3}{7} \right) ^\beta \right] + 2 \cdot \left( \frac{3}{7} \right) ^\beta$
Anthrace	$12 \cdot \left( \frac{1}{6} \right) ^\beta + 8 \cdot \left[ \left( \frac{1}{6} \right) ^\beta + \left( \frac{3}{11} \right) ^\beta \right] + 4 \cdot \left( \frac{3}{11} \right) ^\beta$
Phenanthrene	$14 \cdot \left( \frac{1}{6} \right) ^\beta + 6 \cdot \left[ \left( \frac{1}{6} \right) ^\beta + \left( \frac{3}{11} \right) ^\beta \right] + 6 \cdot \left( \frac{3}{11} \right) ^\beta$
Tetracene	$12 \cdot \left( \frac{1}{8} \right) ^\beta + 12 \cdot \left[ \left( \frac{1}{8} \right) ^\beta + \left( \frac{1}{5} \right) ^\beta \right] + 6 \cdot \left( \frac{1}{5} \right) ^\beta$
Benzo[c]phenanthrene	$16 \cdot \left( \frac{1}{8} \right) ^\beta + 8 \cdot \left[ \left( \frac{1}{8} \right) ^\beta + \left( \frac{1}{5} \right) ^\beta \right] + 10 \cdot \left( \frac{1}{5} \right) ^\beta$
Benzo[a]anthracene	$14 \cdot \left( \frac{1}{8} \right) ^\beta + 10 \cdot \left[ \left( \frac{1}{8} \right) ^\beta + \left( \frac{1}{5} \right) ^\beta \right] + 8 \cdot \left( \frac{1}{5} \right) ^\beta$
Chrysene	$16 \cdot \left( \frac{1}{8} \right) ^\beta + 8 \cdot \left[ \left( \frac{1}{8} \right) ^\beta + \left( \frac{1}{5} \right) ^\beta \right] + 10 \cdot \left( \frac{1}{5} \right) ^\beta$
Triphenylene	$18 \cdot \left( \frac{1}{8} \right) ^\beta + 6 \cdot \left[ \left( \frac{1}{8} \right) ^\beta + \left( \frac{1}{5} \right) ^\beta \right] + 12 \cdot \left( \frac{1}{5} \right) ^\beta$
Pyrene	$12 \cdot \left( \frac{1}{8} \right) ^\beta + 8 \cdot \left[ \left( \frac{1}{8} \right) ^\beta + \left( \frac{1}{5} \right) ^\beta \right] + 10 \cdot \left( \frac{1}{5} \right) ^\beta$
Pentacene	$12 \cdot \left( \frac{1}{10} \right) ^\beta + 16 \cdot \left[ \left( \frac{1}{10} \right) ^\beta + \left( \frac{3}{19} \right) ^\beta \right] + 8 \cdot \left( \frac{3}{19} \right) ^\beta$
Benzo[a]tetracene	$14 \cdot \left( \frac{1}{10} \right) ^\beta + 14 \cdot \left[ \left( \frac{1}{10} \right) ^\beta + \left( \frac{3}{19} \right) ^\beta \right] + 10 \cdot \left( \frac{3}{19} \right) ^\beta$
Dibenzo[a,h]anthracene	$16 \cdot \left( \frac{1}{10} \right) ^\beta + 12 \cdot \left[ \left( \frac{1}{10} \right) ^\beta + \left( \frac{3}{19} \right) ^\beta \right] + 12 \cdot \left( \frac{3}{19} \right) ^\beta$
Dibenzo[a,j]anthracene	$16 \cdot \left( \frac{1}{10} \right) ^\beta + 12 \cdot \left[ \left( \frac{1}{10} \right) ^\beta + \left( \frac{3}{19} \right) ^\beta \right] + 12 \cdot \left( \frac{3}{19} \right) ^\beta$
Pentaphene	$14 \cdot \left( \frac{1}{10} \right) ^\beta + 14 \cdot \left[ \left( \frac{1}{10} \right) ^\beta + \left( \frac{3}{19} \right) ^\beta \right] + 10 \cdot \left( \frac{3}{19} \right) ^\beta$
Benzo[g]chrysene	$20 \cdot \left( \frac{1}{10} \right) ^\beta + 8 \cdot \left[ \left( \frac{1}{10} \right) ^\beta + \left( \frac{3}{19} \right) ^\beta \right] + 16 \cdot \left( \frac{3}{19} \right) ^\beta$
Pentahelicene	$18 \cdot \left( \frac{1}{10} \right) ^\beta + 10 \cdot \left[ \left( \frac{1}{10} \right) ^\beta + \left( \frac{3}{19} \right) ^\beta \right] + 14 \cdot \left( \frac{3}{19} \right) ^\beta$
Benzo[c]chrysene	$16 \cdot \left( \frac{1}{10} \right) ^\beta + 12 \cdot \left[ \left( \frac{1}{10} \right) ^\beta + \left( \frac{3}{19} \right) ^\beta \right] + 12 \cdot \left( \frac{3}{19} \right) ^\beta$
Picene	$18 \cdot \left( \frac{1}{10} \right) ^\beta + 10 \cdot \left[ \left( \frac{1}{10} \right) ^\beta + \left( \frac{3}{19} \right) ^\beta \right] + 14 \cdot \left( \frac{3}{19} \right) ^\beta$
Benzo[b]chrysene	$16 \cdot \left( \frac{1}{10} \right) ^\beta + 12 \cdot \left[ \left( \frac{1}{10} \right) ^\beta + \left( \frac{3}{19} \right) ^\beta \right] + 12 \cdot \left( \frac{3}{19} \right) ^\beta$
Dibenzo[a,c]anthracene	$18 \cdot \left( \frac{1}{10} \right) ^\beta + 10 \cdot \left[ \left( \frac{1}{10} \right) ^\beta + \left( \frac{3}{19} \right) ^\beta \right] + 14 \cdot \left( \frac{3}{19} \right) ^\beta$
Dibenzo[b,g]phenanthrene	$16 \cdot \left( \frac{1}{10} \right) ^\beta + 10 \cdot \left[ \left( \frac{1}{10} \right) ^\beta + \left( \frac{3}{19} \right) ^\beta \right] + 12 \cdot \left( \frac{3}{19} \right) ^\beta$
Perylene	$16 \cdot \left( \frac{1}{9} \right) ^\beta + 8 \cdot \left[ \left( \frac{1}{9} \right) ^\beta + \left( \frac{3}{17} \right) ^\beta \right] + 16 \cdot \left( \frac{3}{17} \right) ^\beta$
Benzo[e]pyrene	$16 \cdot \left( \frac{1}{9} \right) ^\beta + 8 \cdot \left[ \left( \frac{1}{9} \right) ^\beta + \left( \frac{3}{17} \right) ^\beta \right] + 16 \cdot \left( \frac{3}{17} \right) ^\beta$
Benzo[a]pyrene	$14 \cdot \left( \frac{1}{9} \right) ^\beta + 10 \cdot \left[ \left( \frac{1}{9} \right) ^\beta + \left( \frac{3}{17} \right) ^\beta \right] + 14 \cdot \left( \frac{3}{17} \right) ^\beta$
Hexahelicene	$12 \cdot \left( \frac{1}{11} \right) ^\beta + 12 \cdot \left[ \left( \frac{1}{11} \right) ^\beta + \left( \frac{1}{7} \right) ^\beta \right] + 24 \cdot \left( \frac{1}{7} \right) ^\beta$
Benzo[ghi]perylene	$14 \cdot \left( \frac{1}{10} \right) ^\beta + 10 \cdot \left[ \left( \frac{1}{10} \right) ^\beta + \left( \frac{3}{19} \right) ^\beta \right] + 20 \cdot \left( \frac{3}{19} \right) ^\beta$
Hexacene	$12 \cdot \left( \frac{1}{12} \right) ^\beta + 20 \cdot \left[ \left( \frac{1}{12} \right) ^\beta + \left( \frac{3}{23} \right) ^\beta \right] + 10 \cdot \left( \frac{3}{23} \right) ^\beta$
Coronene	$12 \cdot \left( \frac{1}{11} \right) ^\beta + 12 \cdot \left[ \left( \frac{1}{11} \right) ^\beta + \left( \frac{1}{7} \right) ^\beta \right] + 24 \cdot \left( \frac{1}{7} \right) ^\beta$
Ovalene	$8 \cdot \left( \frac{1}{15} \right) ^\beta + 16 \cdot \left[ \left( \frac{1}{15} \right) ^\beta + \left( \frac{3}{29} \right) ^\beta \right] + 38 \cdot \left( \frac{3}{29} \right) ^\beta$

## Optimization formulation and pseudo code

Given $\rho (\alpha )$, the function representing the correlation coefficient between $E_{\pi }$ (Pi Electronic Energy) and two sets of temperature indices $T_{\alpha }^{1}$ and $T_{\alpha }^{2}$, we aim to find the optimal values of $\alpha$ for which the correlation coefficient is maximized. Additionally, we want to identify the range of $\alpha$ values where $T_{\alpha }^{1}$ yields a stronger correlation with $E_{\pi }$ compared to $T_{\alpha }^{2}$. The objective is to maximize $\rho (\alpha )$ with respect to $\alpha$ for both $T_{\alpha }^{1}$ and $T_{\alpha }^{2}$, with the constraint that $\alpha$ must remain within a specified range. Mathematical Foundation is given as follows:

Let $R(\alpha )=R_{\alpha }(Y,X)$ be the correlation function between $Y\in \{C_p,S^o\}$ and $X\in \{R_{\alpha },SCI_{\alpha }\}$. Then, we formulate the following optimization problem:

25 $$\begin{aligned} \begin{aligned} \min _{\alpha } \quad&|R_{\alpha }(Y,X)|\\ \text {s.t.} \quad&0\le |R(\alpha )|\le 1\\&\alpha _{\min }<\alpha <\alpha _{\max } \\&\text {Optimal Values} = \alpha _{\max _1}~\text {for}~T^1_\alpha \\&\text {Optimal Values} = \alpha _{\max _2}~\text {for}~T^2_\alpha \\ \end{aligned} \end{aligned}$$

Next, we present the pseudo-code corresponding to the above optimization formulation.

**Algorithm 1:**
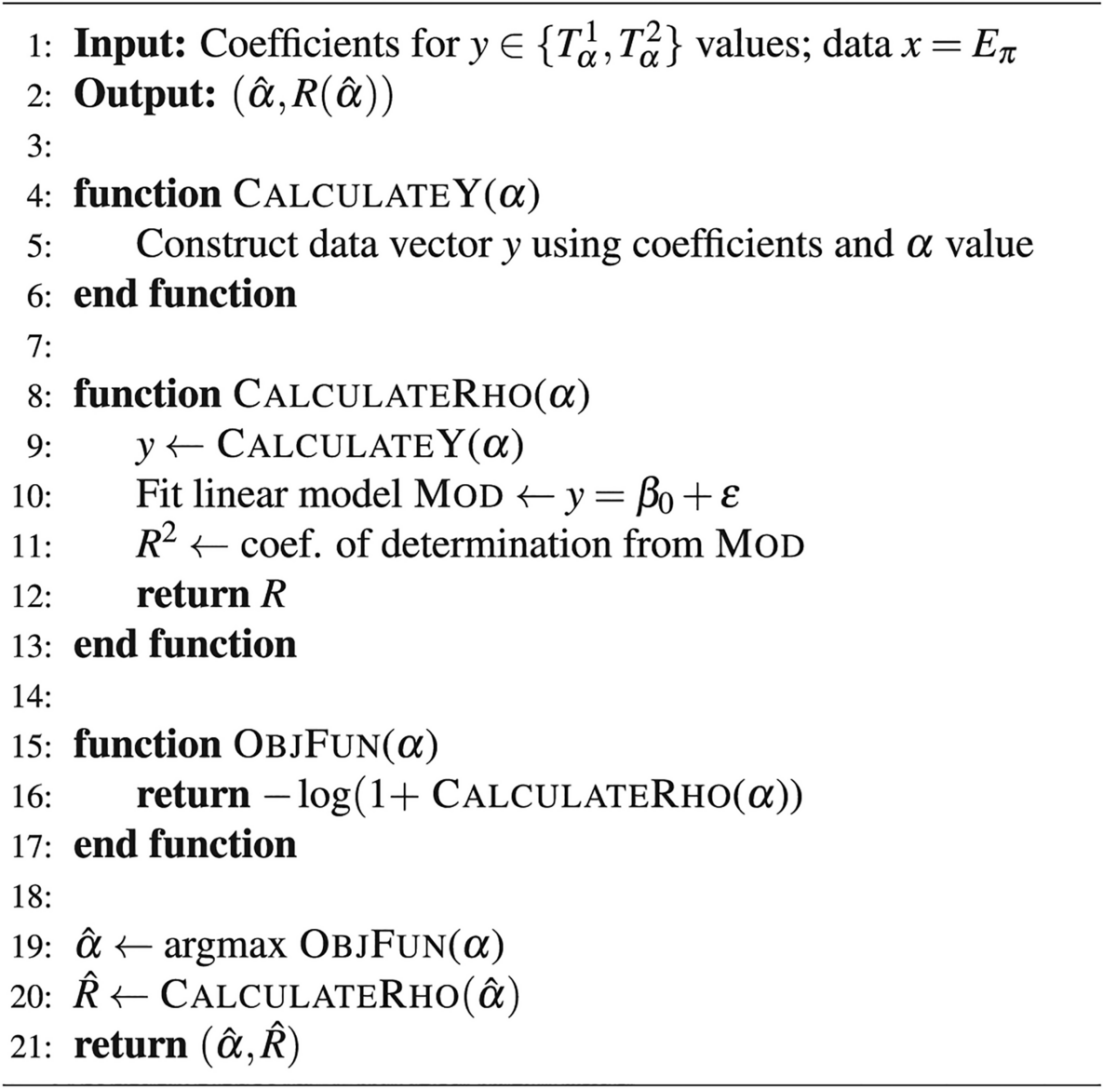
Optimization of correlation function.

## Results and discussion

Consider that the Pi-Electronic $(E_\pi )$ for the lower benzenoid hydrocarbons (BHs) may be predicted with a high degree of accuracy in a temperature range of $\beta$ using the *F*-temperature index $T_{\beta }$.

Using the technique outlined in Section 3, we first calculate the precise analytical formulas for $T_{\beta }$ for the bottom 30 BHs listed in Table 1. Specifically, to calculate their precise values, we use the formulas for $T_{\beta }$ in (24), respectively. It is important to note that the $T_{\beta }$ values of a particular hexagonal system can only be calculated using the number of vertices *v*, inlets *k*, and hexagons *p*. The *F*-temperature index for a particular BH graph is computed using the methods described in Section 3, as demonstrated by the following example.

### Example 5.1

The graph of Phenanthrene, denoted as *P*, is shown in Table 1. Next, *P* is made up of two fissures, one bay, three hexagons, and fourteen vertices. Consequently, $p=3$, $k=3$, and $v=14$. In (24), with these values, deliver:

$$\begin{aligned} T_\beta (P)= & 14 \cdot \left( \frac{1}{6} \right) ^\beta + 6 \cdot \left[ \left( \frac{1}{6} \right) ^\beta + \left( \frac{3}{11} \right) ^\beta \right] + 6 \cdot \left( \frac{3}{11} \right) ^\beta . \end{aligned}$$

Table 2 contains the data that we obtain by applying this approach to every graph in Table 1.

**Fig. 4:**
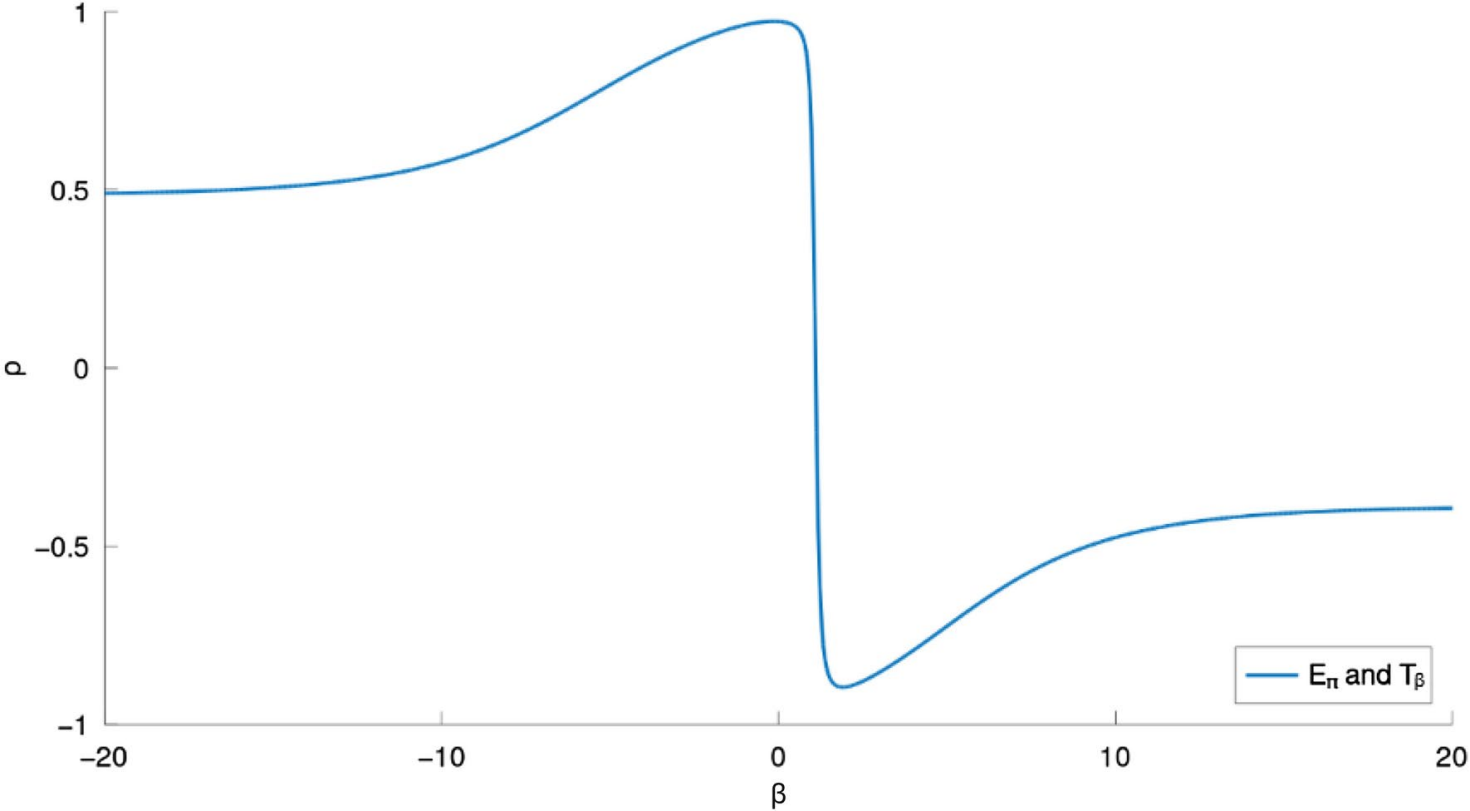
Correlation coefficient curves between general indices and $E_\pi$ of lower benzenoids (far-view). Generated by implementing Algorithm 1 on the computational platform Octave.

**Fig. 5:**
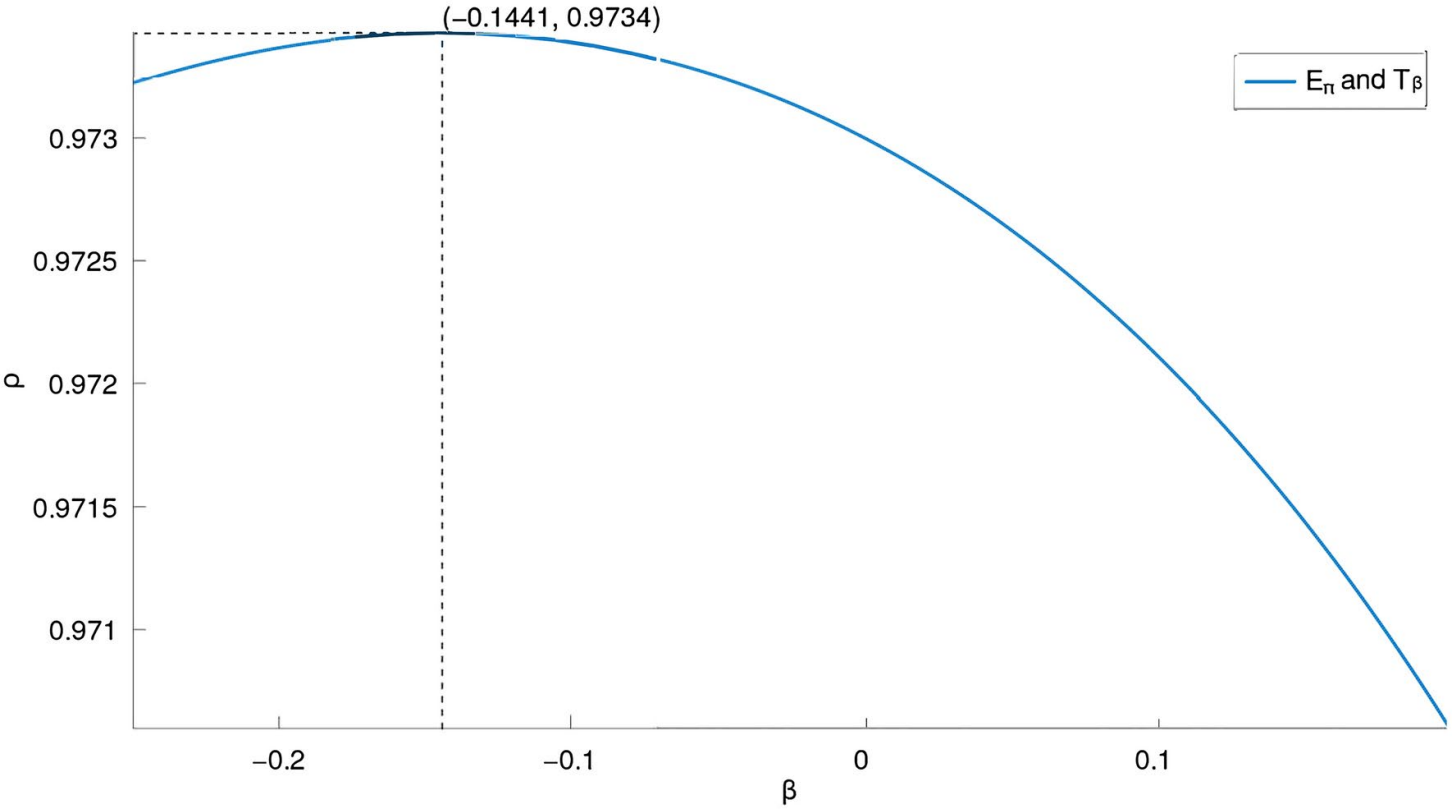
Curves of correlation coefficients relating general indices to $E_\pi$ of lower benzenoids. Generated by implementing Algorithm 1 on the computational platform Octave.

We created two curves from the data in Table 2, which are depicted in Figures 4 and 5. The physicochemical property ($E_\pi$ in 4 & 5) and index ($T_{\beta }$) correlation coefficient curves for these 30 lower BHs are displayed in the corresponding figures as solid lines that are color-coded.

The *F*-temperature index $T_{\beta }$, as seen in Figure 5, is the best estimate of Pi-Electronic $E_{\pi }$ for BHs for $\beta \in (-0.1441, 0)$ when compared to the other general indices.

While $\beta$ is within a certain interval, there is a strong connection between $E_\pi$ and $T_\beta$. For $\beta$ at other intervals, a similar strong connection is also present.

**Fig. 6:**
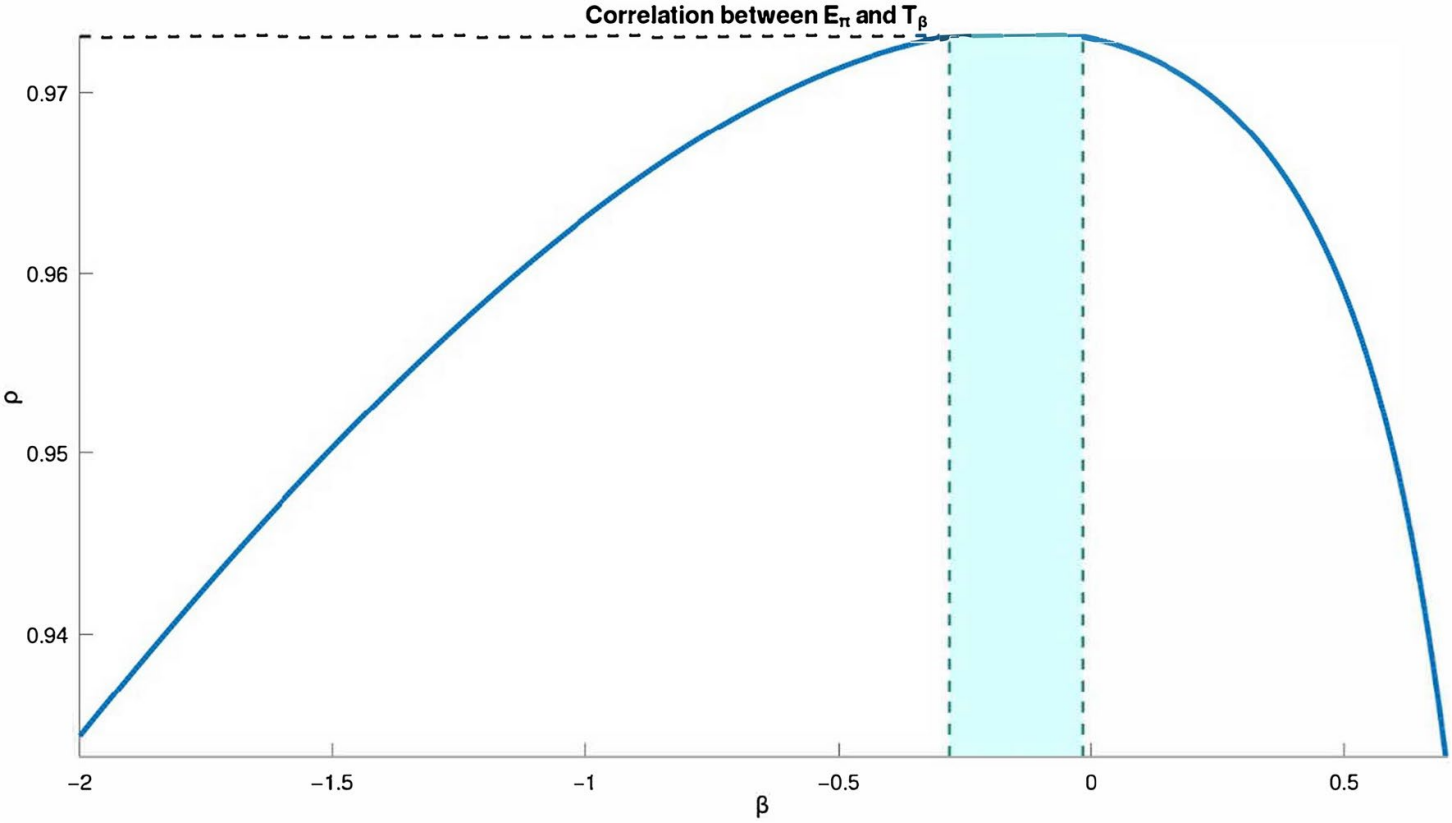
Ranges of good $\rho$ for $E_\pi$-$T_\beta$ for smaller BHs are presented in Fig. 6. The graph in Fig. 6 was generated by implementing Algorithm 1 on the computational platform Octave.

It can be shown from Figure 5 that, for a set of 30 lower BHs, $T_{-0.1441}$ is the most linearly correlated product-connectivity index with $E_\pi$ among all of them, and $T_{-0.1441}$ is the most linearly correlated sum-connectivity index with $E_\pi$ for all of them. The physicochemical characteristics ($E_\pi$) and the two aforementioned indices, respectively, have the following linear relationships (with 95% confidence intervals):

26 $$\begin{aligned} \begin{aligned}&E_\pi = 3.421_{\pm 0.765} - 0.196_{\pm 0.006} \hspace{0.25em} T_{-0.1441}, \\ \rho (&bp, T_{-0.1441}) = 0.997086439, s(bp,T_{-0.1441}) = 0.550638079, \end{aligned} \end{aligned}$$

**Fig. 7:**
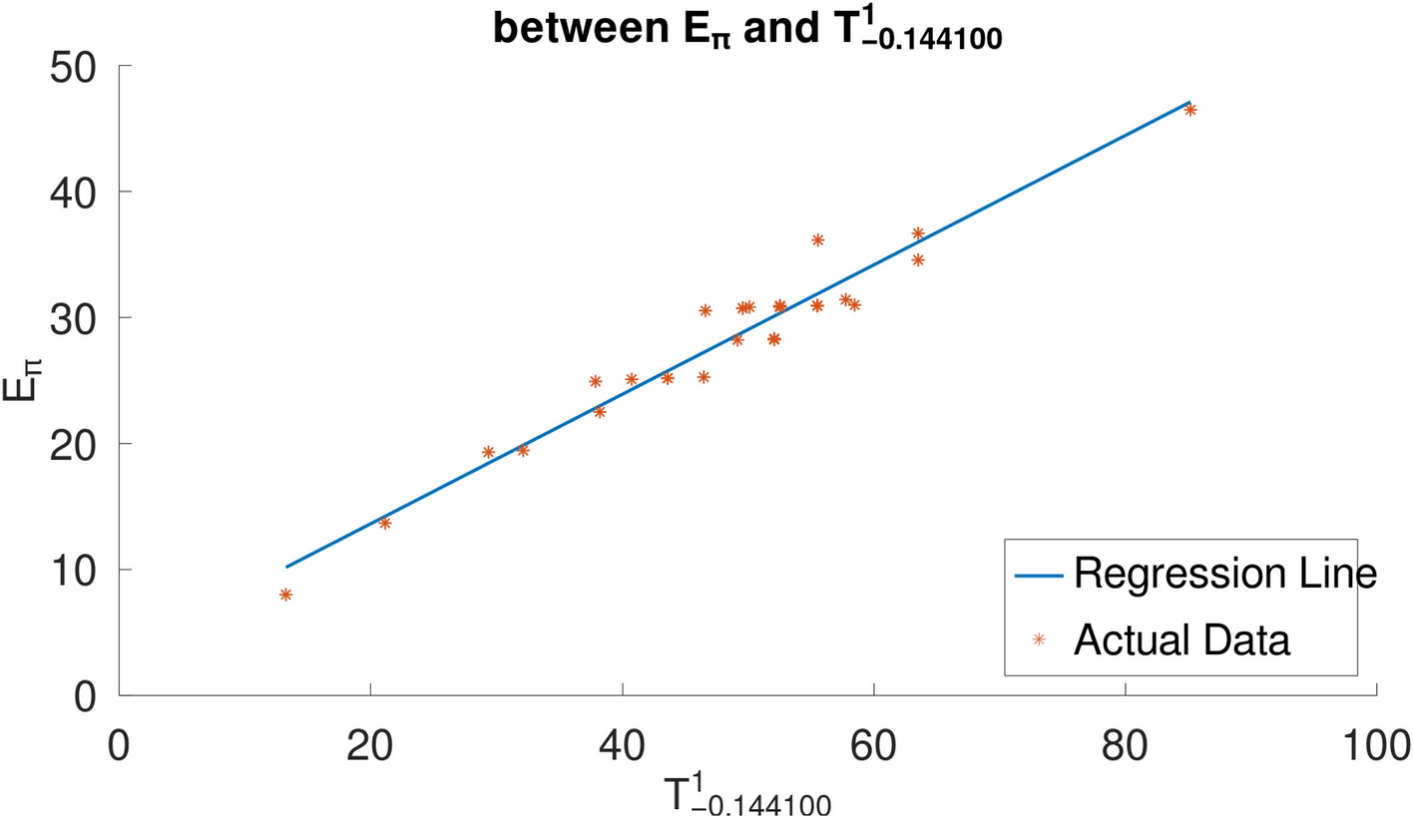
Scatter plots of $E_\pi$-$T_{-0.1441}$, for lower benzenoids. Generated by implementing Algorithm 1 on the computational platform Octave.

The standard error of fit and correlation coefficient are represented by the values *s* and $\rho$, respectively. Scatter plots between the indices $T_{-0.1441}$ and the Pi-Electronic $E_\pi$ for the 30 lower benzenoids are displayed in Figure 7.

As can be seen from 26, the two product-connectivity indices that perform the best at determining the Pi Electronic Energy are $T^1_{-0.094882}$ and $T^2_{-0.046133}$, respectively.

## Computational results of carbon nanocones based on MatLab programming

This section provides a computational approach that uses computer software to compute specific graphical indices related to temperature. This computing method requires the simultaneous use of computer-based software programs like MatLab, etc. to work. High-performance programming language for computational technology is called MatLab. It incorporates programming, computation, and visualization into an intuitive setting where problems are expressed and their solutions are conveyed via standard mathematical symbols. Graphs may be efficiently transformed into arrays and combinations by MatLab, which is then used for specific graphical studies. Mathematical techniques such as processing pictures and videos in computational graphics, mechanical and civil engineering, and more employ matrices in the Matlab software.

The technique that follows generates particular temperature-based graphical indices in Section 2 given a chemical graph $\vartheta$ as input.

**Step 1**: Determine the edge partitioning of $\vartheta$ by drawing it.

**Step 2**: Insert the appropriate partitioning into our MatLab code to compute any temperature-based index from Section 2.

A GitHub public repository has been established, comprising a ReadMe file and the Matlab code. You can reach the repository using https://github.com/saimaafazal/Temperature-based-Indices-of-Carbon-Nanocones.

**Fig. 8:**
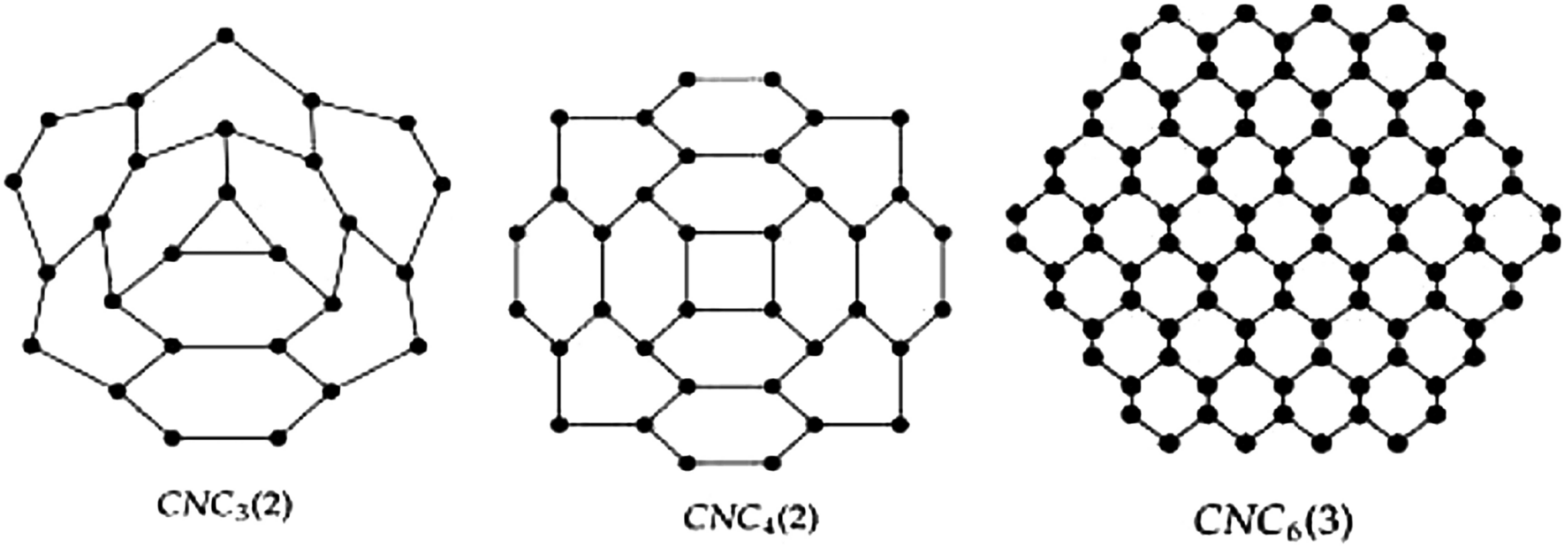
Some members of carbon nanocone. These images are generated by using Mayura draw.

We verify our calculations in the following Tables 3 and 4 by using our proposed method for some nanocones. Some members of carbon nanocones are shown in Figure 8.

**Table 3:** Temperature Indices of Carbon Nanocone $CNC_{k}[n]$.

$CNC_{k}[n]$	$HT_1$	*ST*	$HT_2$	*PT*	*RPT*	*AGT*	*FT*
$CNC_{3}[1]$	4.85	20.31	0.11	56.24	4.15	15.20	2.48
$CNC_{3}[2]$	1.89	76.00	0.01	325.50	4.07	36.30	0.96
$CNC_{4}[1]$	3.15	32.35	0.03	106.73	3.87	20.23	1.60
$CNC_{4}[2]$	1.34	118.68	0.0024	594.80	3.95	48.38	0.68
$CNC_{5}[1]$	2.32	46.09	0.01	173.08	3.72	25.27	1.18
$CNC_{5}[2]$	1.04	167.23	0.0012	944.49	3.89	60.46	0.52
$CNC_{6}[1]$	1.83	61.34	0.01	255.30	3.63	30.31	0.93
$CNC_{6}[2]$	0.84	221.03	0.0006	1374.57	3.84	72.54	0.43
$CNC_{7}[1]$	1.51	77.97	0.0043	353.38	3.56	35.35	0.77
$CNC_{7}[2]$	0.71	279.60	0.0004	1885.05	3.81	84.63	0.36

**Table 4:** Temperature Indices of Carbon Nanocone $CNC_{k}[n]$.

$CNC_{k}[n]$	*TSO*	$^mTSO$	*HT*	*GAT*	*RRPT*	*ABCT*
$CNC_{3}[1]$	6.01	38.77	55.50	14.81	10.78	67.90
$CNC_{3}[2]$	5.83	226.17	322.57	35.71	31.90	433.88
$CNC_{4}[1]$	5.59	73.79	105.49	19.78	16.08	135.85
$CNC_{4}[2]$	5.67	413.64	589.71	47.63	44.02	806.22
$CNC_{5}[1]$	5.37	119.84	171.21	24.74	21.24	226.18
$CNC_{5}[2]$	5.57	657.14	936.65	59.55	56.09	1292.23
$CNC_{6}[1]$	5.23	176.93	252.67	29.70	26.33	338.92
$CNC_{6}[2]$	5.51	956.67	1363.40	71.47	68.13	1891.93
$CNC_{7}[1]$	5.13	245.06	349.85	34.66	31.40	474.09
$CNC_{7}[2]$	5.46	1312.23	1869.94	83.39	80.16	2605.32

## Computational results for carbon nanocone $CNC_{k}[n]$

**Fig. 9:**
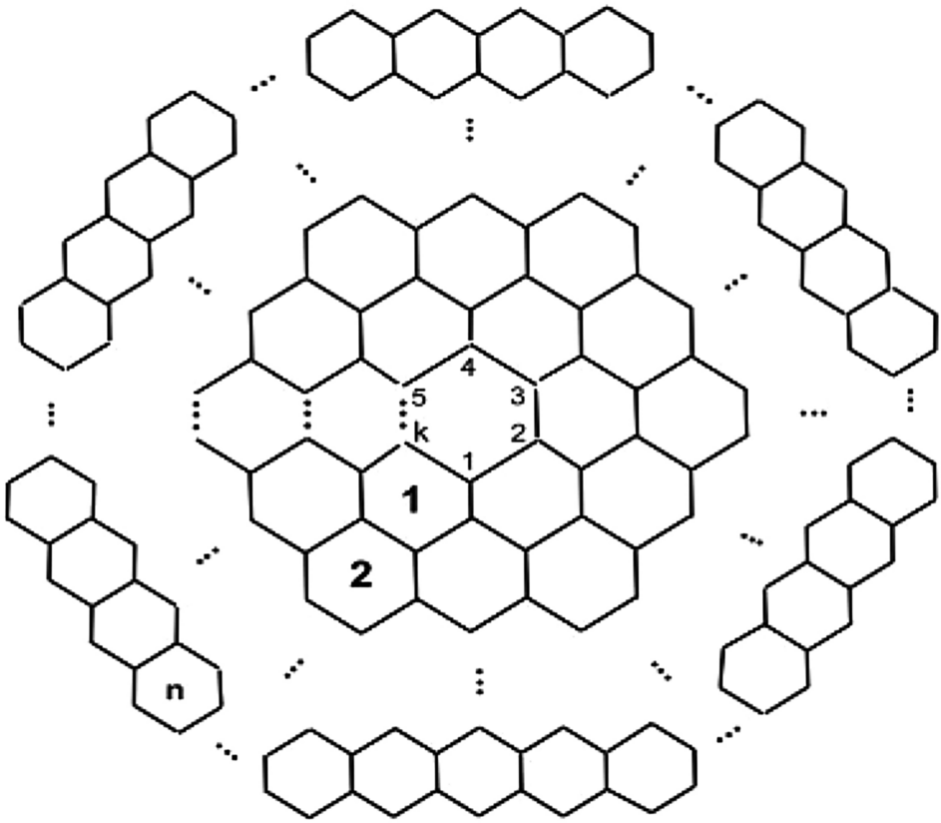
Carbon nanocone $CNC_{k}[n]$. This image is generated by using Mayura draw.

In this section, we use edge partitioning to derive the topological indices of the carbon nanocone $CNC_{k}[n]$, which are dependent on the graph’s vertices’ temperatures. Initially, we display the $CNC_{k}[n]$ graph, as illustrated by Figure 9.

The edge partitioning of the provided graph is represented by $(T_r, T_s )$ for any arbitrary vertices *r* and *s* of $CNC_{k}[n]$. This is further explained in the Table 5 that follows. We denote $T_r$ and $T_s$ for temperature index of vertex *r* and *s* respectively.

**Table 5:** Edge Partition of Carbon Nanocone $CNC_{k}[n]$.

$(T_r, T_s)\setminus rs \in E(G)$	*Number of edges*
$\left( \frac{2}{k\sum _{i=1}^{n+1}(2i-1)-2}, \frac{2}{k\sum _{i=1}^{n+1}(2i-1)-2}\right)$	*k*
$\left( \frac{2}{k\sum _{i=1}^{n+1}(2i-1)-2}, \frac{3}{k\sum _{i=1}^{n+1}(2i-1)-3}\right)$	2*kn*
$\left( \frac{3}{k\sum _{i=1}^{n+1}(2i-1)-3}, \frac{3}{k\sum _{i=1}^{n+1}(2i-1)-3}\right)$	$k\sum _{i=1}^{n}(3i-1)$

### Theorem 7.1

*The carbon nanocone* $CNC_{k}[n]$ *has general first temperature index*

27 $$\begin{aligned} T^\sigma _1(CNC_{k}[n])&=k\left( \frac{4}{k\sum _{i=1}^{n+1}(2i-1)-2}\right) ^\sigma + 2kn\left( \frac{5k\sum _{i=1}^{n+1}(2i-1)-12}{\left( k\sum _{i=1}^{n+1}(2i-1)\right) ^2-5\sum _{i=1}^{n+1}(2i-1)+6}\right) ^\sigma \nonumber \\&\quad + k\sum _{i=1}^{n}(3i-1)\left( \frac{6}{k\sum _{i=1}^{n+1}(2i-1)-3} \right) ^\sigma . \end{aligned}$$

### *Proof*

Let $CNC_{k}[n]$ be a carbon nanocone. By definition of $T^\sigma _1$, we have

$$\begin{aligned} T^\sigma _1(CNC_{k}[n])=\sum _{edges} (T_r+ T_s)^\sigma . \end{aligned}$$

then utilizing edge partitioning in the Table 5, we arrive at

$$\begin{aligned} T^\sigma _1(CNC_{k}[n])&= |E_1|\left( \frac{2}{k\sum _{i=1}^{n+1}(2i-1)-2} + \frac{2}{k\sum _{i=1}^{n+1}(2i-1)-2}\right) ^\sigma \\&\quad +|E_2|\left( \frac{2}{k\sum _{i=1}^{n+1}(2i-1)-2} + \frac{3}{k\sum _{i=1}^{n+1}(2i-1)-3}\right) ^\sigma + |E_3|\left( \frac{3}{k\sum _{i=1}^{n+1}(2i-1)-3} + \frac{3}{k\sum _{i=1}^{n+1}(2i-1)-3}\right) ^\sigma \\ T^\sigma _1(CNC_{k}[n])&= k\left( \frac{4}{k\sum _{i=1}^{n+1}(2i-1)-2}\right) ^\sigma +2kn\left( \frac{5k\sum _{i=1}^{n+1}(2i-1)-12}{\left( k\sum _{i=1}^{n+1}(2i-1)\right) ^2-5\sum _{i=1}^{n+1}(2i-1)+6}\right) ^\sigma \\&\quad + k\sum _{i=1}^{n}(3i-1)\left( \frac{6}{k\sum _{i=1}^{n+1}(2i-1)-3} \right) ^\sigma . \end{aligned}$$

$$\square$$

### Corollary 7.2

*The first hyper temperature index of carbon nanocone* $CNC_{k}[n]$ *is*

$$\begin{aligned} HT_1(CNC_{k}[n])&=\frac{16k}{\left( k\sum _{i=1}^{n+1}(2i-1)-2\right) ^2} + 2kn\left( \frac{5k\sum _{i=1}^{n+1}(2i-1)-12}{\left( k\sum _{i=1}^{n+1}(2i-1)\right) ^2-5\sum _{i=1}^{n+1}(2i-1)+6}\right) ^2 \\&\quad +\frac{36k\sum _{i=1}^{n}(3i-1)}{\left( k\sum _{i=1}^{n+1}(2i-1)-3 \right) ^2}. \end{aligned}$$

### Corollary 7.3

*The sum-connectivity temperature index of carbon nanocone* $CNC_{k}[n]$ *is*

$$\begin{aligned} ST(CNC_{k}[n])&=\frac{k}{2}\left( k\sum _{i=1}^{n+1}(2i-1)-2\right) ^{\frac{1}{2}} + 2kn\left( \frac{5k\sum _{i=1}^{n+1}(2i-1)-12}{\left( k\sum _{i=1}^{n+1}(2i-1)\right) ^2-5\sum _{i=1}^{n+1}(2i-1)+6}\right) ^{-\frac{1}{2}} \\&\quad + \frac{k}{\sqrt{6}}\sum _{i=1}^{n}(3i-1)\left( k\sum _{i=1}^{n+1}(2i-1)-3 \right) ^{\frac{1}{2}}. \end{aligned}$$

### *Proof*

When $\sigma = 2 \ \& \ -\frac{1}{2}$ is entered into Equation 27, the previously stated outcomes are obtained.

### Theorem 7.4

*The carbon nanocone* $CNC_{k}[n]$ *has general second temperature index*

$$\begin{aligned} T^\sigma _2(CNC_{k}[n]&=k\left( \frac{2}{k\sum _{i=1}^{n+1}(2i-1)-2}\right) ^{2\sigma } + 2kn\left( \frac{6}{\left( k\sum _{i=1}^{n+1}(2i-1)\right) ^2-5\sum _{i=1}^{n+1}(2i-1)+6}\right) ^\sigma \\&\quad + k\sum _{i=1}^{n}(3i-1)\left( \frac{3}{k\sum _{i=1}^{n+1}(2i-1)-3}\right) ^{2\sigma }. \end{aligned}$$

### *Proof*

Let $CNC_{k}[n]$ be a carbon nanocone. By definition of $T^\sigma _2$, we have

$$\begin{aligned} T^\sigma _2(CNC_{k}[n] )=\sum _{edges} (T_r T_s)^\sigma . \end{aligned}$$

then utilizing edge partitioning in the table 5, we arrive at

28 $$\begin{aligned} T^\sigma _2(CNC_{k}[n])&= |E_1|\left( \frac{2}{k\sum _{i=1}^{n+1}(2i-1)-2} \times \frac{2}{k\sum _{i=1}^{n+1}(2i-1)-2}\right) ^\sigma \nonumber \\&\quad + |E_2|\left( \frac{2}{k\sum _{i=1}^{n+1}(2i-1)-2} \times \frac{3}{k\sum _{i=1}^{n+1}(2i-1)-3}\right) ^\sigma + |E_3|\left( \frac{3}{k\sum _{i=1}^{n+1}(2i-1)-3} \times \frac{3}{k\sum _{i=1}^{n+1}(2i-1)-3}\right) ^\sigma \nonumber \\ T^\sigma _2(CNC_{k}[n])&=k\left( \frac{2}{k\sum _{i=1}^{n+1}(2i-1)-2}\right) ^{2\sigma } + 2kn\left( \frac{6}{\left( k\sum _{i=1}^{n+1}(2i-1)\right) ^2-5\sum _{i=1}^{n+1}(2i-1)+6}\right) ^\sigma \nonumber \\&\quad + k\sum _{i=1}^{n}(3i-1)\left( \frac{3}{k\sum _{i=1}^{n+1}(2i-1)-3}\right) ^{2\sigma }. \end{aligned}$$

### Corollary 7.5

*The second hyper temperature index of carbon nanocone* $CNC_{k}[n]$ *is*

$$\begin{aligned} HT_2(CNC_{k}[n])&=\frac{16k}{\left( k\sum _{i=1}^{n+1}(2i-1)-2\right) ^{4}} + \frac{72kn}{\left( \left( k\sum _{i=1}^{n+1}(2i-1)\right) ^2-5\sum _{i=1}^{n+1}(2i-1)+6\right) ^2} \\&\quad + \frac{81k\sum _{i=1}^{n}(3i-1)}{\left( k\sum _{i=1}^{n+1}(2i-1)-3\right) ^{4}}. \end{aligned}$$

### Corollary 7.6

*The product-connectivity temperature index of carbon nanocone* $CNC_{k}[n]$ *is*

$$\begin{aligned} PT(CNC_{k}[n])&=\frac{k}{2}\left( k\sum _{i=1}^{n+1}(2i-1)-2\right) + \frac{2kn}{\sqrt{6}}\left( \left( k\sum _{i=1}^{n+1}(2i-1)\right) ^2-5\sum _{i=1}^{n+1}(2i-1)+6\right) ^{\frac{1}{2}} \\&\quad + \frac{k}{3}\sum _{i=1}^{n}(3i-1)\left( k\sum _{i=1}^{n+1}(2i-1)-3\right) . \end{aligned}$$

### Corollary 7.7

*The reciprocal Product-connectivity temperature index of carbon nanocone* $CNC_{k}[n]$ *is*

$$\begin{aligned} RPT(CNC_{k}[n])&=\frac{2k}{k\sum _{i=1}^{n+1}(2i-1)-2} + \frac{2\sqrt{6}kn}{\left( \left( k\sum _{i=1}^{n+1}(2i-1)\right) ^2-5\sum _{i=1}^{n+1}(2i-1)+6\right) ^{\frac{1}{2}}} \\&\quad + \frac{3k\sum _{i=1}^{n}(3i-1)}{k\sum _{i=1}^{n+1}(2i-1)-3}. \end{aligned}$$

### *Proof*

Equation 28 yields the required result when $\sigma = 2,\ -\frac{1}{2} \ \& \ \frac{1}{2}$ is entered.

### Theorem 7.8

*The arithmetic-geometric temperature index of carbon nanocone* $CNC_{k}[n]$ *is*

$$\begin{aligned} AGT(CNC_{k}[n])=k\left( 1 + \sum _{i=1}^{n}(3i-1) + \frac{5kn\sum _{i=1}^{n+1}(2i-1)-12n}{\sqrt{6\left( k\sum _{i=1}^{n+1}(2i-1)\right) ^2-30\sum _{i=1}^{n+1}(2i-1)+36}}\right) . \end{aligned}$$

### *Proof*

Let $CNC_{k}[n]$ be a carbon nanocone. By definition of *AGT*, we have

$$\begin{aligned} AGT(CNC_{k}[n])= \sum _{edges} \frac{T_r+ T_s}{2\sqrt{T_r T_s}}. \end{aligned}$$

then utilizing edge partitioning in the Table 5, we arrive at

$$\begin{aligned} AGT( CNC_{k}[n])&= |E_1|\left( \frac{\frac{2}{k\sum _{i=1}^{n+1}(2i-1)-2} + \frac{2}{k\sum _{i=1}^{n+1}(2i-1)-2}}{2\sqrt{\left( \frac{2}{k\sum _{i=1}^{n+1}(2i-1)-2}\right) \left( \frac{2}{k\sum _{i=1}^{n+1}(2i-1)-2}\right) }}\right) \\&\quad + |E_2|\left( \frac{\frac{2}{k\sum _{i=1}^{n+1}(2i-1)-2} + \frac{3}{k\sum _{i=1}^{n+1}(2i-1)-3}}{2\sqrt{\left( \frac{2}{k\sum _{i=1}^{n+1}(2i-1)-2}\right) \left( \frac{3}{k\sum _{i=1}^{n+1}(2i-1)-3}\right) }}\right) + |E_3|\left( \frac{\frac{3}{k\sum _{i=1}^{n+1}(2i-1)-3} + \frac{3}{k\sum _{i=1}^{n+1}(2i-1)-3}}{2\sqrt{\left( \frac{3}{k\sum _{i=1}^{n+1}(2i-1)-3}\right) \left( \frac{3}{k\sum _{i=1}^{n+1}(2i-1)-3}\right) }}\right) \end{aligned}$$

$$\begin{aligned} AGT(CNC_{k}[n])&= k \left( \frac{\frac{4}{k\sum _{i=1}^{n+1}(2i-1)-2}}{2\sqrt{\left( \frac{2}{k\sum _{i=1}^{n+1}(2i-1)-2}\right) ^2}}\right) + 2kn\left( \frac{\frac{5k\sum _{i=1}^{n+1}(2i-1)-12}{\left( k\sum _{i=1}^{n+1}(2i-1)\right) ^2-5\sum _{i=1}^{n+1}(2i-1)+6}}{2\sqrt{\frac{6}{ \left( k\sum _{i=1}^{n+1}(2i-1)\right) ^2-5\sum _{i=1}^{n+1}(2i-1)+6}}}\right) \\&\quad + k\sum _{i=1}^{n}(3i-1)\left( \frac{\frac{6}{k\sum _{i=1}^{n+1}(2i-1)-3}}{2\sqrt{\left( \frac{3}{k\sum _{i=1}^{n+1}(2i-1)-3}\right) ^2}}\right) . \end{aligned}$$

$$\begin{aligned} AGT(CNC_{k}[n]) = k + \left( \frac{5k^2n\sum _{i=1}^{n+1}(2i-1)-12kn}{\sqrt{6\left( k\sum _{i=1}^{n+1}(2i-1)\right) ^2-30\sum _{i=1}^{n+1}(2i-1)+36}}\right) + k\sum _{i=1}^{n}(3i-1) \end{aligned}$$

$$\begin{aligned} AGT(CNC_{k}[n]) = k\left( 1 + \sum _{i=1}^{n}(3i-1) + \frac{5kn\sum _{i=1}^{n+1}(2i-1)-12n}{\sqrt{6\left( k\sum _{i=1}^{n+1}(2i-1)\right) ^2-30\sum _{i=1}^{n+1}(2i-1)+36}}\right) . \end{aligned}$$

$$\square$$  

### Theorem 7.9

*The general temperature index of carbon nanocone* $CNC_{k}[n]$ *is*

$$\begin{aligned} T_\sigma (CNC_{k}[n])= 2k(1+n)\left( \frac{2}{k\sum _{i=1}^{n+1}(2i-1)-2}\right) ^\sigma + 2k(n+\sum _{i=1}^{n}(3i-1))\left( \frac{3}{k\sum _{i=1}^{n+1}(2i-1)-3}\right) ^\sigma . \end{aligned}$$

### *Proof*

Let $CNC_{k}[n]$ be a carbon nanocone. By definition of $T_\sigma$, we have

$$\begin{aligned} T_\sigma ( CNC_{k}[n])= \sum _{edges}(T_r^\sigma + T_s^\sigma ). \end{aligned}$$

then utilizing edge partitioning in the Table 5, we arrive at

$$\begin{aligned} T_\sigma (CNC_{k}[n])&= |E_1|\left( \left( \frac{2}{k\sum _{i=1}^{n+1}(2i-1)-2}\right) ^\sigma + \left( \frac{2}{k\sum _{i=1}^{n+1}(2i-1)-2}\right) ^\sigma \right) \\&\quad + |E_2|\left( \left( \frac{2}{k\sum _{i=1}^{n+1}(2i-1)-2}\right) ^\sigma + \left( \frac{3}{k\sum _{i=1}^{n+1}(2i-1)-3}\right) ^\sigma \right) \\&\quad + |E_3|\left( \left( \frac{3}{k\sum _{i=1}^{n+1}(2i-1)-3}\right) ^\sigma + \left( \frac{3}{k\sum _{i=1}^{n+1}(2i-1)-3}\right) ^\sigma \right) . \end{aligned}$$

$$\begin{aligned} T_\sigma (CNC_{k}[n])&= k\left( 2\left( \frac{2}{k\sum _{i=1}^{n+1}(2i-1)-2}\right) ^\sigma \right) \\&\quad + 2kn\left( \left( \frac{2}{k\sum _{i=1}^{n+1}(2i-1)-2}\right) ^\sigma + \left( \frac{3}{k\sum _{i=1}^{n+1}(2i-1)-3}\right) ^\sigma \right) + k\sum _{i=1}^{n}(3i-1) \left( 2\left( \frac{3}{k\sum _{i=1}^{n+1}(2i-1)-3}\right) ^\sigma \right) \end{aligned}$$

29 $$\begin{aligned} T_\sigma (CNC_{k}[n])= 2k(1+n)\left( \frac{2}{k\sum _{i=1}^{n+1}(2i-1)-2}\right) ^\sigma + 2k(n+\sum _{i=1}^{n}(3i-1))\left( \frac{3}{k\sum _{i=1}^{n+1}(2i-1)-3}\right) ^\sigma . \end{aligned}$$

$$\square$$

### Corollary 7.10

*The F-temperature index of carbon nanocones* $CNC_{k}[n]$ *is*

$$\begin{aligned} FT(CNC_{k}[n])=\frac{8k(1+n)}{\left( k\sum _{i=1}^{n+1}(2i-1)-2\right) ^2} + \frac{18k\left( n+\sum _{i=1}^{n}(3i-1)\right) }{\left( k\sum _{i=1}^{n+1}(2i-1)-3\right) ^2}. \end{aligned}$$

### *Proof*

Equation 29 yields the expected answer when $\sigma =2$ is entered. $\square$

### Theorem 7.11

*The temperature Somber index of carbon nanocone* $CNC_{k}[n]$ *is*

$$\begin{aligned} TSO(CNC_{k}[n])&=\frac{2\sqrt{2}k}{k\sum _{i=1}^{n+1}(2i-1)-2} + 2kn \left( \frac{\sqrt{13\left( k\sum _{i=1}^{n+1}(2i-1)\right) ^2 -60k\sum _{i=1}^{n+1}(2i-1)+72}}{\left( k\sum _{i=1}^{n+1}(2i-1)\right) ^2-5\sum _{i=1}^{n+1}(2i-1)+6}\right) \\&\quad + \frac{3\sqrt{2}k\sum _{i=1}^{n}(3i-1)}{k\sum _{i=1}^{n+1}(2i-1)-3}. \end{aligned}$$

### *Proof*

Let $CNC_{k}[n]$ be a carbon nanocone. By definition of *TSO*, we have

$$\begin{aligned} TSO(CNC_{k}[n])= \sum _{edges} \sqrt{T_r^2+ T_s^2}. \end{aligned}$$

then utilizing edge partitioning in the Table 5, we arrive at

$$\begin{aligned} TSO(CNC_{k}[n])&= |E_1|\left( \sqrt{\left( \frac{2}{k\sum _{i=1}^{n+1}(2i-1)-2}\right) ^2 + \left( \frac{2}{k\sum _{i=1}^{n+1}(2i-1)-2}\right) ^2}\right) \\&\quad + |E_2|\left( \sqrt{\left( \frac{2}{k\sum _{i=1}^{n+1}(2i-1)-2}\right) ^2 + \left( \frac{3}{k\sum _{i=1}^{n+1}(2i-1)-3}\right) ^2}\right) \\&\quad + |E_3|\left( \sqrt{\left( \frac{3}{k\sum _{i=1}^{n+1}(2i-1)-3}\right) ^2 + \left( \frac{3}{k\sum _{i=1}^{n+1}(2i-1)-3}\right) ^2}\right) ,\\ TSO(CNC_{k}[n])&= k\left( \sqrt{2\left( \frac{2}{k\sum _{i=1}^{n+1}(2i-1)-2}\right) ^2}\right) \\&\quad +2kn \left( \sqrt{\frac{4}{(k\sum _{i=1}^{n+1}(2i-1)-2)^2} + \frac{9}{(k\sum _{i=1}^{n+1}(2i-1)-3)^2}}\right) + k\sum _{i=1}^{n}(3i-1)\left( \sqrt{2\left( \frac{3}{k\sum _{i=1}^{n+1}(2i-1)-3}\right) ^2}\right) \\ TSO(CNC_{k}[n])&= \frac{2\sqrt{2}k}{k\sum _{i=1}^{n+1}(2i-1)-2} + 2kn \left( \frac{\sqrt{\left( 2k\sum _{i=1}^{n+1}(2i-1)-6\right) ^2 + \left( 3k\sum _{i=1}^{n+1}(2i-1)-6\right) ^2}}{\left( k\sum _{i=1}^{n+1}(2i-1)\right) ^2-5\sum _{i=1}^{n+1}(2i-1)+6}\right) \\&\quad + \frac{3\sqrt{2}k\sum _{i=1}^{n}(3i-1)}{k\sum _{i=1}^{n+1}(2i-1)-3},\\ TSO(CNC_{k}[n])&= \frac{2\sqrt{2}k}{k\sum _{i=1}^{n+1}(2i-1)-2} + 2kn \left( \frac{\sqrt{13\left( k\sum _{i=1}^{n+1}(2i-1)\right) ^2 -60k\sum _{i=1}^{n+1}(2i-1)+72}}{\left( k\sum _{i=1}^{n+1}(2i-1)\right) ^2-5\sum _{i=1}^{n+1}(2i-1)+6}\right) \\&\quad +\frac{3\sqrt{2}k\sum _{i=1}^{n}(3i-1)}{k\sum _{i=1}^{n+1}(2i-1)-3}. \end{aligned}$$

$$\square$$

### Theorem 7.12

*The modified temperature Sombor index of carbon nanocone* $CNC_{k}[n]$ *is*

$$\begin{aligned} ^{m}TSO(CNC_{k}[n])&=\left( \frac{k^2\sum _{i=1}^{n+1}(2i-1)-2k}{\sqrt{2^3}}\right) + 2kn\left( \frac{\left( k\sum _{i=1}^{n+1}(2i-1)\right) ^2-5\sum _{i=1}^{n+1}(2i-1)+6}{\sqrt{\left( 2k\sum _{i=1}^{n+1}(2i-1)-6\right) ^2 + \left( 3k\sum _{i=1}^{n+1}(2i-1)-6\right) ^2}}\right) \\&\quad + k\sum _{i=1}^{n}(3i-1)\left( \frac{k\sum _{i=1}^{n+1}(2i-1)-3}{3\sqrt{2}}\right) . \end{aligned}$$

### *Proof*

Let $CNC_{k}[n]$ be a carbon nanocone. By definition of $^{m}TSO$, we have

$$\begin{aligned} ^{m}TSO(CNC_{k}[n])= \sum _{edges}\frac{1}{\sqrt{T_r^2+ T_s^2}}. \end{aligned}$$

then utilizing edge partitioning in the Table 5, we arrive at

$$\begin{aligned} ^{m}TSO(CNC_{k}[n])&= |E_1|\left( \frac{1}{\sqrt{\left( \frac{2}{k\sum _{i=1}^{n+1}(2i-1)-2}\right) ^2 + \left( \frac{2}{k\sum _{i=1}^{n+1}(2i-1)-2}\right) ^2}}\right) +\\&\quad + |E_2|\left( \frac{1}{\sqrt{\left( \frac{2}{k\sum _{i=1}^{n+1}(2i-1)-2}\right) ^2 + \left( \frac{3}{k\sum _{i=1}^{n+1}(2i-1)-3}\right) ^2}}\right) + |E_3|\left( \frac{1}{\sqrt{\left( \frac{3}{k\sum _{i=1}^{n+1}(2i-1)-3}\right) ^2 + \left( \frac{3}{k\sum _{i=1}^{n+1}(2i-1)-3}\right) ^2}}\right) ,\\ ^{m}TSO(CNC_{k}[n])&= \left( \frac{k^2\sum _{i=1}^{n+1}(2i-1)-2k}{\sqrt{2^3}}\right) \\&\quad + 2kn\left( \frac{\left( k\sum _{i=1}^{n+1}(2i-1)\right) ^2-5\sum _{i=1}^{n+1}(2i-1)+6}{\sqrt{\left( 2k\sum _{i=1}^{n+1}(2i-1)-6\right) ^2 + \left( 3k\sum _{i=1}^{n+1}(2i-1)-6\right) ^2}}\right) + k\sum _{i=1}^{n}(3i-1)\left( \frac{k\sum _{i=1}^{n+1}(2i-1)-3}{3\sqrt{2}}\right) . \end{aligned}$$

$$\square$$

### Theorem 7.13

*The harmonic temperature index of carbon nanocone* $CNC_{k}[n]$ *is*

$$\begin{aligned} HT(CNC_{k}[n])&= k\left( \frac{k\sum _{i=1}^{n+1}(2i-1)-3}{2}\right) + 4kn\left( \frac{\left( k\sum _{i=1}^{n+1}(2i-1)\right) ^2-5\sum _{i=1}^{n+1}(2i-1)+6}{5k\sum _{i=1}^{n+1}(2i-1) -12}\right) \\&\quad + k\sum _{i=1}^{n}(3i-1) \left( \frac{k\sum _{i=1}^{n+1}(2i-1)-3}{3}\right) . \end{aligned}$$

### *Proof*

Let $CNC_{k}[n]$ be a carbon nanocone. By definition of *HT*, we have

$$\begin{aligned} HT(CNC_{k}[n])= \sum _{edges} \frac{2}{T_r+ T_s}. \end{aligned}$$

then utilizing edge partitioning in the Table 5, we arrive at

$$\begin{aligned} HT(CNC_{k}[n])&= |E_1|\left( \frac{2}{\frac{2}{k\sum _{i=1}^{n+1}(2i-1)-2} + \frac{2}{k\sum _{i=1}^{n+1}(2i-1)-2}}\right) + |E_2|\left( \frac{2}{\frac{2}{k\sum _{i=1}^{n+1}(2i-1)-2} + \frac{3}{k\sum _{i=1}^{n+1}(2i-1)-3}}\right) \\&\quad + |E_3|\left( \frac{2}{\frac{3}{k\sum _{i=1}^{n+1}(2i-1)-3} + \frac{3}{k\sum _{i=1}^{n+1}(2i-1)-3}}\right) ,\\ HT(CNC_{k}[n])&= k\left( \frac{k\sum _{i=1}^{n+1}(2i-1)-2}{2}\right) + 4kn\left( \frac{\left( k\sum _{i=1}^{n+1}(2i-1)\right) ^2-5\sum _{i=1}^{n+1}(2i-1)+6}{5k\sum _{i=1}^{n+1}(2i-1) -12}\right) \\&\quad + k\sum _{i=1}^{n}(3i-1) \left( \frac{k\sum _{i=1}^{n+1}(2i-1)-3}{3}\right) . \end{aligned}$$

### Theorem 7.14

*The geometric-Arithematic temperature index of carbon nanocone* $CNC_{k}[n]$ *is*

$$\begin{aligned} GAT(CNC_{k}[n])=k + 4kn\left( \frac{\sqrt{6(k\sum _{i=1}^{n+1}(2i-1)-2)(k\sum _{i=1}^{n+1}(2i-1)-3)}}{5k\sum _{i=1}^{n+1}(2i-1)-12}\right) + k\sum _{i=1}^{n}(3i-1). \end{aligned}$$

### *Proof*

Let $CNC_{k}[n]$ be a carbon nanocone. By definition of *GAT*, we have

$$\begin{aligned} GAT(CNC_{k}[n])= \sum _{edges}\left( \frac{2\sqrt{T_r T_s}}{T_r+ T_s}\right) . \end{aligned}$$

then utilizing edge partitioning in the Table 5, we arrive at

$$\begin{aligned} GAT(CNC_{k}[n])&= |E_1|\left( \frac{2 \sqrt{\left( \frac{2}{k\sum _{i=1}^{n+1}(2i-1)-2}\right) \left( \frac{2}{k\sum _{i=1}^{n+1}(2i-1)-2}\right) }}{\frac{2}{k\sum _{i=1}^{n+1}(2i-1)-2} + \frac{2}{k\sum _{i=1}^{n+1}(2i-1)-2}}\right) \\&\quad + |E_2|\left( \frac{2 \sqrt{\left( \frac{2}{k\sum _{i=1}^{n+1}(2i-1)-2}\right) \left( \frac{3}{k\sum _{i=1}^{n+1}(2i-1)-3}\right) }}{\frac{2}{k\sum _{i=1}^{n+1}(2i-1)-2} + \frac{3}{k\sum _{i=1}^{n+1}(2i-1)-3}}\right) + |E_3|\left( \frac{2\sqrt{\left( \frac{3}{k\sum _{i=1}^{n+1}(2i-1)-3}\right) \left( \frac{3}{k\sum _{i=1}^{n+1}(2i-1)-3}\right) }}{\frac{3}{k\sum _{i=1}^{n+1}(2i-1)-3} + \frac{3}{k\sum _{i=1}^{n+1}(2i-1)-3}} \right) ,\\ GAT(CNC_{k}[n])&= k\left( \frac{2\sqrt{\left( \frac{2}{k\sum _{i=1}^{n+1}(2i-1)-2}\right) ^2}}{\frac{4}{k\sum _{i=1}^{n+1}(2i-1)-2}}\right) + 2kn\left( \frac{2 \sqrt{\frac{6}{\left( k\sum _{i=1}^{n+1}(2i-1)\right) ^2-5\sum _{i=1}^{n+1}(2i-1)+6}}}{\frac{5k\sum _{i=1}^{n+1}(2i-1)-12}{\left( k\sum _{i=1}^{n+1}(2i-1)\right) ^2- 5\sum _{i=1}^{n+1}(2i-1)+6}}\right) \\&\quad + k\sum _{i=1}^{n}(3i-1)\left( \frac{2\sqrt{\left( \frac{3}{k\sum _{i=1}^{n+1}(2i-1)-3}\right) ^2}}{\frac{6}{k\sum _{i=1}^{n+1}(2i-1)-3}} \right) . \end{aligned}$$

$$\begin{aligned} GAT(CNC_{k}[n])= k + 4kn\left( \frac{\sqrt{6\left( k\sum _{i=1}^{n+1}(2i-1)\right) ^2-30\sum _{i=1}^{n+1}(2i-1)+36}}{5k\sum _{i=1}^{n+1}(2i-1)-12}\right) + k\sum _{i=1}^{n}(3i-1). \end{aligned}$$

$$\square$$

### Theorem 7.15

*The reduced reciprocal product-connectivity temperature index of carbon nanocone* $CNC_{k}[n]$ *is*

$$\begin{aligned} RRPT(CNC_{k}[n])&=k\left( \frac{4-k\sum _{i=1}^{n+1}(2i-1)}{k\sum _{i=1}^{n+1}(2i-1)-2}\right) + 2kn \sqrt{\left( \frac{(k\sum _{i=1}^{n+1}(2i-1))^2 -10k\sum _{i=1}^{n+1}(2i-1)+ 24}{\left( k\sum _{i=1}^{n+1}(2i-1)\right) ^2-5\sum _{i=1}^{n+1}(2i-1)+6}\right) } \\&\quad + k\sum _{i=1}^{n}(3i-1)\left( \frac{6-k\sum _{i=1}^{n+1}(2i-1)}{k\sum _{i=1}^{n+1}(2i-1)-3}\right) . \end{aligned}$$

### *Proof*

Let $CNC_{k}[n]$ be a carbon nanocone. By definition of *RRPT*, we have

$$\begin{aligned} RRPT(CNC_{k}[n])= \sum _{edges}\sqrt{(T_r-1)( T_s-1)}. \end{aligned}$$

then utilizing edge partitioning in the Table 5, we arrive at

$$\begin{aligned} RRPT(CNC_{k}[n])&= |E_1|\left( \sqrt{\left( \frac{2}{k\sum _{i=1}^{n+1}(2i-1)-2}-1\right) \left( \frac{2}{k\sum _{i=1}^{n+1}(2i-1)-2}-1\right) } \right) \\&\quad + |E_2|\left( \sqrt{\left( \frac{2}{k\sum _{i=1}^{n+1}(2i-1)-2}-1\right) \left( \frac{3}{k\sum _{i=1}^{n+1}(2i-1)-3}-1\right) } \right) \\&\quad + |E_3|\left( \sqrt{\left( \frac{3}{k\sum _{i=1}^{n+1}(2i-1)-3}-1\right) \left( \frac{3}{k\sum _{i=1}^{n+1}(2i-1)-3}-1\right) } \right) ,\\ RRPT(CNC_{k}[n])&= k\sqrt{\left( \frac{4-k\sum _{i=1}^{n+1}(2i-1)}{k\sum _{i=1}^{n+1}(2i-1)-2}\right) ^2} + 2kn \sqrt{\left( \frac{4-k\sum _{i=1}^{n+1}(2i-1)}{k\sum _{i=1}^{n+1}(2i-1)-2}\right) \left( \frac{6-k\sum _{i=1}^{n+1}(2i-1)}{k\sum _{i=1}^{n+1}(2i-1)-3}\right) }\\&\quad + k\sum _{i=1}^{n}(3i-1) \sqrt{\left( \frac{6-k\sum _{i=1}^{n+1}(2i-1)}{k\sum _{i=1}^{n+1}(2i-1)-3}\right) ^2},\\ RRPT(CNC_{k}[n])&= k\left( \frac{4-k\sum _{i=1}^{n+1}(2i-1)}{k\sum _{i=1}^{n+1}(2i-1)-2}\right) + 2kn \sqrt{\left( \frac{(k\sum _{i=1}^{n+1}(2i-1))^2 -10k\sum _{i=1}^{n+1}(2i-1)+ 24}{\left( k\sum _{i=1}^{n+1}(2i-1)\right) ^2-5\sum _{i=1}^{n+1}(2i-1)+6}\right) } \\&\quad + k\sum _{i=1}^{n}(3i-1)\left( \frac{6-k\sum _{i=1}^{n+1}(2i-1)}{k\sum _{i=1}^{n+1}(2i-1)-3}\right) . \end{aligned}$$

$$\square$$

### Theorem 7.16

*The atom-bond connectivity temperature index of carbon nanocone* $CNC_{k}[n]$ *is*

$$\begin{aligned} ABCT(CNC_{k}[n])&=k\left( \frac{\sqrt{2(k\sum _{i=1}^{n+1}(2i-1))^2 -12k\sum _{i=1}^{n+1}(2i-1) + 16}}{2}\right) \\&\quad +2kn\left( \sqrt{\frac{2(k\sum _{i=1}^{n+1}(2i-1))^2 - 15k\sum _{i=1}^{n+1}(2i-1)+ 24)}{6}}\right) \\&\quad + k\sum _{i=1}^{n}(3i-1) \left( \frac{\sqrt{2(k\sum _{i=1}^{n+1}(2i-1))^2 -18k\sum _{i=1}^{n+1}(2i-1) + 36}}{3}\right) . \end{aligned}$$

### *Proof*

Let $CNC_{k}[n]$ be a carbon nanocone. By definition of *ABCT*, we have

$$\begin{aligned} ABCT(CNC_{k}[n])= \sum _{edges} \sqrt{\left| \frac{T_r + T_s -2}{T_r T_s}\right| }. \end{aligned}$$

then utilizing edge partitioning in the Table 5, we arrive at

$$\begin{aligned} ABCT(CNC_{k}[n])&= |E_1|\left( \sqrt{\left| \frac{\frac{2}{k\sum _{i=1}^{n+1}(2i-1)-2}+ \frac{2}{k\sum _{i=1}^{n+1}(2i-1)-2}-2}{\left( \frac{2}{k\sum _{i=1}^{n+1}(2i-1)-2}\right) \left( \frac{2}{k\sum _{i=1}^{n+1}(2i-1)-2}\right) } \right| }\right) + |E_2|\left( \sqrt{\left| \frac{\frac{2}{k\sum _{i=1}^{n+1}(2i-1)-2}+ \frac{3}{k\sum _{i=1}^{n+1}(2i-1)-3}-2}{\left( \frac{2}{k\sum _{i=1}^{n+1}(2i-1)-2}\right) \left( \frac{3}{k\sum _{i=1}^{n+1}(2i-1)-3}\right) } \right| }\right) \\&\quad + |E_3|\left( \sqrt{\left| \frac{\frac{3}{k\sum _{i=1}^{n+1}(2i-1)-3}+ \frac{3}{k\sum _{i=1}^{n+1}(2i-1)-3}-2}{\left( \frac{3}{k\sum _{i=1}^{n+1}(2i-1)-3}\right) \left( \frac{3}{k\sum _{i=1}^{n+1}(2i-1)-3}\right) } \right| }\right) ,\\ ABCT(CNC_{k}[n])&= k\left( \sqrt{\left| \frac{\frac{8-2k\sum _{i=1}^{n+1}(2i-1)}{k\sum _{i=1}^{n+1}(2i-1)-2}}{\left( \frac{2}{k\sum _{i=1}^{n+1}(2i-1)-2}\right) ^2} \right| }\right) + 2kn\left( \sqrt{\left| \frac{-\left( 2(k\sum _{i=1}^{n+1}(2i-1))^2-15k\sum _{i=1}^{n+1}(2i-1) + 24\right) }{6} \right| }\right) \\&\quad + k\sum _{i=1}^{n}(3i-1) \left( \sqrt{\left| \frac{\frac{12-2k\sum _{i=1}^{n+1}(2i-1)}{k\sum _{i=1}^{n+1}(2i-1)-3}}{\left( \frac{3}{k\sum _{i=1}^{n+1}(2i-1)-3}\right) ^2} \right| }\right) ,\\ ABCT(CNC_{k}[n])&= k\left( \frac{\sqrt{2(k\sum _{i=1}^{n+1}(2i-1))^2 -12k\sum _{i=1}^{n+1}(2i-1) + 16}}{2}\right) \\&\quad + 2kn\left( \sqrt{\frac{2(k\sum _{i=1}^{n+1}(2i-1))^2 - 15k\sum _{i=1}^{n+1}(2i-1)+ 24)}{6}}\right) \\&\quad + k\sum _{i=1}^{n}(3i-1) \left( \frac{\sqrt{2(k\sum _{i=1}^{n+1}(2i-1))^2 -18k\sum _{i=1}^{n+1}(2i-1) + 36}}{3}\right) . \end{aligned}$$

$$\square$$

## Conclusions

This research demonstrates that, for benzenoid hydrocarbons, there is a positive connection in some intervals (see Figure 6) between the Pi Electronic ($E_\pi$) and the *F*-temperature index ($T_\beta$). Moreover, for $\beta \in (-0.1441,0)$ (marked with blue dashed lines in 5, $T_\beta$ is the best measure of Pi Electronic of *G*. Also, in this paper, we reached at closed formula of temperature-based topological indices for $CNC_{k}[n], k\ge 3, n\in N$ nanocones. We found that the temperatures of the end vertices for each $CNC_{k}[n]$ nanocone caused the edge set to be partitioned. We used these partitions to compute $HT_{1}$ , $HT_{2}$, *ST*, *PT*, *RPT*, *AGT*, *FT*, *TSO*, $^mTSO$, *HT*, *GAT*, *RRPT* and *ABCT* indices of $CNC_{k}[n], k\ge 3, n\in N$ nanocones by using MatLab code. A ReadMe file and MatLab code for calculating the temperature-based indices of carbon nanocones have also been added to our public GitHub repository.

## Future work

This study assessed the general product-connectivity index’s and general sum-connectivity index’s correlation power to calculate the boiling point (*bp*) and enthalpy of formation ($\Delta H^0_f$) of benzenoid hydrocarbons, respectively. We suggest doing more research to investigate additional generalized valency-based indices in a similar manner in order to ascertain *bp* and $\Delta H^0_f$ of hydrocarbons benzenoid. We also propose calculations for the temperature indices of some other chemical graph and their graphical representation. Also propose the entropy measure of temperature-based indices for chemical graph such as carbon nanocones.

## Data Availability

The datasets generated and/or analysed during the current study are available in the GitHub repository, https://github.com/NoorazamTuah/Predective_Potential_FTemp_Indices.
